# Microbial community networks across body sites are associated with susceptibility to respiratory infections in infants

**DOI:** 10.1038/s42003-021-02755-1

**Published:** 2021-10-28

**Authors:** Marta Reyman, Melanie Clerc, Marlies A. van Houten, Kayleigh Arp, Mei Ling J. N. Chu, Raiza Hasrat, Elisabeth A. M. Sanders, Debby Bogaert

**Affiliations:** 1 Department of Paediatric Immunology and Infectious Diseases, Wilhelmina Children’s Hospital, University Medical Centre Utrecht, Utrecht, The Netherlands; 2 Spaarne Gasthuis Academy, Hoofddorp and Haarlem, The Netherlands; 3 Centre for Inflammation Research, Queen’s Medical Research Institute, University of Edinburgh, Edinburgh, UK; 4 National Institute for Public Health and the Environment, Bilthoven, The Netherlands; 5 Present Address: Department of Dermatology, Erasmus Medical Center, Rotterdam, The Netherlands; 6 Present Address: Institute of Microbiology, ETH Zürich, Zürich, Switzerland

**Keywords:** Microbiome, Microbial ecology

## Abstract

Respiratory tract infections are a major cause of morbidity and mortality worldwide in young children. Concepts such as the gut-lung axis have highlighted the impact of microbial communities at distal sites in mediating disease locally. However, little is known about the extent to which microbial communities from multiple body sites are linked, and how this relates to disease susceptibility. Here, we combine 16S-based rRNA sequencing data from 112 healthy, term born infants, spanning three body sites (oral cavity, nasopharynx, gut) and the first six months of life. Using a cross-niche microbial network approach, we show that, already from the first week of life on, there is a strong association between both network structure and species essential to these structures (hub species), and consecutive susceptibility to respiratory tract infections in this cohort. Our findings underline the crucial role of cross-niche microbial connections in respiratory health.

## Abstract (web summary)

In order to assess the extent by which microbial communities from multiple body sites are linked and related to disease susceptibility, Reyman and Clerc et al. combined 16S-based rRNA sequencing data from 112 healthy, term born infants, spanning three body sites (oral cavity, nasopharynx, gut) over the first six months of life. They demonstrate a strong association between network structure and species and susceptibility to respiratory tract infections, suggesting a crucial role of cross-niche microbial connections in respiratory health.

## Introduction

The human microbiome is widely recognised as an important mediator of health and disease, making it the subject of extensive study. The microbiome is highly variable between individuals but also within one individual when studied over time or across body sites^1^. Exposures to a wide range of environmental factors, such as delivery mode, antibiotics, and diet, have been shown to contribute to this variation^2^. In general, microbiome research has focused on the relation between the microbial community composition of a single anatomical niche and health or disease parameters.

However, it is becoming increasingly clear that direct or indirect effects of microbial communities at distal body sites exist and can also have major implications for both development, as well as severity of a number of diseases locally^3^. One well-established example of cross-niche microbial interaction is the gut-lung axis. It has been shown that microbial components and metabolites in both the gut and lung are capable of modulating immunity not only locally but also systemically^4^. Additionally, specific taxa in both the gastrointestinal and respiratory tract are associated with lung diseases such as asthma, chronic obstructive pulmonary disease, and respiratory tract infections (RTIs)^4^.

Next to variation in species presence/absence and abundance within niches, other features such as the microbial network structure may also contribute to susceptibility to RTIs. Commonly, studies of microbiome networks have been restricted to microbial networks within a single niche^5,6^. For instance, a study performed in patients suffering from inflammatory bowel disease (IBD) showed that the community structure of the microbial network within the gut was distorted in IBD patients^7^. However, it has been hypothesised that the structure of the network formed by the microbes within a specific niche might not only affect local disease development and/or severity but might also have more systemic effects related to disease^8^. Even though examples for such effects are emerging from the literature, we currently lack a conceptual understanding if and to what extent variation in the structure of cross-niche microbial networks might also affect disease susceptibility locally. Understanding these relationships would substantially increase our ability to formulate holistic hypotheses about the development and improvement of integrated diagnostic and treatment approaches for a wide range of diseases.

Here, we, therefore, investigate the structure of microbial networks across different body sites in order to identify signatures in the overall microbial community network in infancy that can be associated with susceptibility to RTIs in the first year of life. To do so, we used 16S rRNA sequencing data from samples of a Dutch cohort of 112 healthy, term born infants collected longitudinally over the first 6 months of life and spanning three body sites (oral cavity, nasopharynx, and gastrointestinal tract). We aimed to (1) compare the development of the microbial communities per niche over time, (2) build cross-niche microbial networks, (3) identify hub species within the networks, and (4) study network structure and hub species in relation to RTI susceptibility during the first year of life. Using this stepwise approach, we found a strong association between cross-niche network structure and hub species and consecutive susceptibility to respiratory tract infections.

## Results

### Niche comparison

Of the 1,250 available samples obtained from the three niches at 1 week, 2, 4, and 6 months of life of 112 healthy infants, 1,248 samples fulfilled our quality threshold (433 faecal samples, 430 nasopharyngeal samples, and 385 saliva samples). Sequencing of these samples resulted in 58,608,834 high quality reads with a minimum Good’s coverage of 99.47% (median 99.97%). The overall Operational Taxonomical Unit (OTU)-table, including the samples of the three niches collected at the four time points, contained 1,148 bacterial OTUs distributed over 18 bacterial phyla, with *Firmicutes* being the most abundant phylum and *Streptococcus* the most abundant genus.

In the oral cavity, the most abundant genus over the first 6 months of life was *Streptococcus*, while this was *Moraxella* for the nasopharynx and *Bifidobacterium* for the gut. Observed species richness was highest in the nasopharyngeal samples, with 895 OTUs identified, compared to 746 OTUs in the saliva and 595 OTUs in the faecal samples. In addition, the nasopharynx also contained the highest number of unique OTUs (defined as observed in a single niche only), namely 232, followed by 121 in the gut and only 38 in the oral cavity. In other words, the oral niche contained a relatively high number of observed species but few unique OTUs, and so it seems to be a reservoir sharing many OTUs with the nasopharynx and gut. Although highly variable in abundance, a total of 331 overlapping OTUs were observed in all three niches, including the top 10 most abundant OTUs in the overall dataset of the combined niches: *Streptococcus* (1), *Bifidobacterium* (2), *Moraxella* (3), *Staphylococcus* (4), *Corynebacterium propinquum* (5), *Streptococcus salivarius* (6), *Dolosigranulum* (7), *Escherichia coli* (9), *Veillonella* (10), and *Haemophilus* (8).

Supplementary Fig. 1 shows the succession patterns of the 15 most abundant OTUs in each niche, highlighting the gradual increase of *Bifidobacterium* (2) over time in the gut (Supplementary Fig. 1a). In the nasopharynx, the initial high abundance of *Staphylococcus* (4) at 1 week of life was gradually replaced by an increasing abundance of *C. propinquum* (5), *Dolosigranulum* (7), *Moraxella* (3), and *Haemophilus* (8) (Supplementary Fig. 1b). Lastly, the figure highlights an overall dominance of *Streptococcus* (1) in the oral cavity (Supplementary Fig. 1c). Observed species richness increased over time in both faecal and saliva samples, though this effect was most pronounced in saliva (linear mixed effect model including age and diversity per niche: *p* < 0.0001 for both niches; Supplementary Fig. 2).

**Fig. 1:**
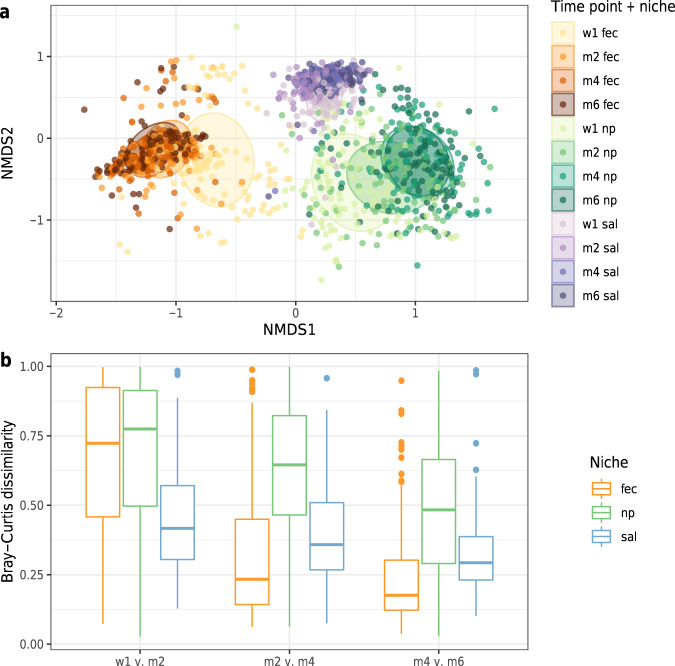
Development of the microbiota community composition for individual niches over time. Non-metric multidimensional scaling (nMDS) plot (**a**), based on Bray–Curtis (BC) dissimilarity between samples, visualising the overall gut microbial community composition stratified for each niche per time point. Each data point represents the microbial community composition of one sample. The ellipses represent the standard deviation of data points belonging to each group, with the centre points of the ellipses calculated using the mean of the coordinates per group. The stress of the ordination was 0.18. In panel **b** the temporal community stability for each niche is shown. As a measure of microbiota stability, we calculated the Bray–Curtis distance between consecutive sample pairs belonging to each individual per time interval, i.e., between week 1 and month 2, between month 2 and month 4, and between month 4 and month 6. Boxplots with medians are shown; the lower and upper hinges correspond to the first and third quartiles (the 25th and 75th percentiles); the upper and lower whiskers extend from the hinge to the largest and smallest value no further than 1.5*IQR from the hinge; outliers are plotted individually. Fec faeces, np nasopharynx, sal saliva. Temporal stability was highest for the saliva samples in early-life, which was later overtaken by a higher stability in consecutive faecal samples (median BC dissimilarity between months 4 and 6 for faecal samples 0.18 versus 0.29 for saliva, Wilcoxon test, *p* < 0.0001).

Microbial community development over time (shown in Fig. 1a) was most stable for the oral cavity in early-life, although this was overtaken by higher stability in the gut at the later time points (median Bray–Curtis [BC] dissimilarity between month 4 and 6 for faecal samples 0.18 versus 0.29 for saliva, Wilcoxon test, *p* < 0.0001; Fig. 1b). The microbial community composition in the nasopharynx showed the lowest temporal stability compared to the other two niches over the first 6 months of life. As expected, the community composition differed significantly between the three niches at all time points, although niche composition was most similar at the earliest time point, in line with the mutual origin of initial microbial seeding at birth. This was followed by a gradual deviation into niche-specific communities over time (calculating the association of niche with composition using PERMANOVA, week 1: *R*^2^ 31.8%, month 2: *R*^2^ 47.8%, month 4; *R*^2^ 57.5%, month 6: *R*^2^ 56.3%, all *p*-values < 0.0001).

Characteristics explaining the overall community composition across niches and time points were in order of importance, niche itself (*R*^2^ 42.4%, adjusted *p*-value 0.00079), followed by age with a much smaller effect size (R^2^ 1.8%, adjusted *p*-value 0.00079), and presence of siblings <5 years, pets, breastfeeding at time of sampling, season of birth, mode of delivery, exposure to antibiotics in the month prior to sampling, day care attendance, pacifier use, and duration of hospital stay after birth (Supplementary Table 1). When studying the niches separately and cross-sectionally per time point, we observed different effect sizes of associations between environmental variables and microbial community composition (Supplementary Table 2). In total, we identified eight covariates to be associated with the nasopharyngeal microbiome (among others presence of siblings <5 years, breastfeeding at sampling and season of birth), seven covariates for the faecal microbiome (among others mode of delivery, breastfeeding at sampling and day care attendance), and only three covariates for the salivary microbiome composition (breastfeeding at sampling, antibiotic use 1 month prior to sampling and season of birth).

### Network analysis

We identified a total of 107 niche indicator OTUs (defined as characteristic, but not exclusive, for a particular niche), with 18 niche indicator OTUs specific for the nasopharynx, 37 for the gut and 52 for the oral cavity. Participants were divided into three groups based on the distribution of the number of RTIs they experienced over the first year of life (0-2 RTIs, 3-4 RTIs, 5–7 RTIs). After selecting the 100 most abundant OTUs per niche and per RTI group, we constructed for each of the three RTI groups a cross-niche microbial network per time point using the SpiecEasi pipeline^9^. We studied the formation of bacterial clusters within these cross-niche networks, and whether clusters were indicative of a specific niche or not, based on the proportion of niche indicator OTUs the clusters contained. In the results below, we will further refer to the cross-niche networks as networks.

**Fig. 2:**
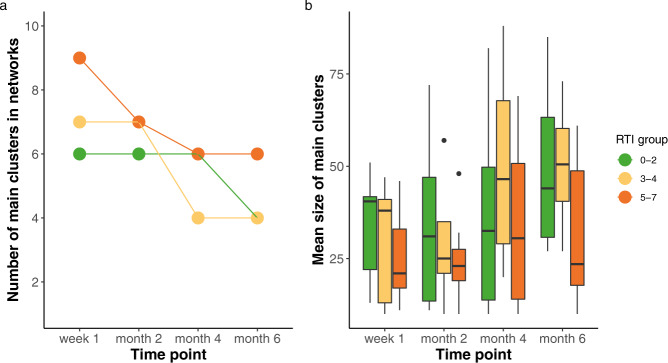
Cluster number and size of the cross-niche microbial networks built per RTI group. Number of main clusters (**a**) and mean size of main clusters (**b**) over time. Number of clusters at week 1 were *n* = 22 (*n* =6 for 0–2 RTIs, *n* = 7 for 3–4 RTIs and *n* = 9 for 5–7 RTIs), *n* = 20 at month 2 (*n* = 6 for 0–2 RTIs, *n* = 7 for 3–4 RTIs and *n* = 7 for 5–7 RTIs), *n* = 16 at month 4 (*n* = 6 for 0–2 RTIs, *n* = 4 for 3–4 RTIs and *n* = 6 for 5–7 RTIs) and *n* = 14 at month 6 (*n* = 4 for 0–2 RTIs, *n* = 4 for 3–4 RTIs and *n* = 6 for 5–7 RTIs). Boxplots with medians are shown; the lower and upper hinges correspond to the first and third quartiles (the 25th and 75th percentiles); the upper and lower whiskers extend from the hinge to the largest and smallest value no further than 1.5 *IQR from the hinge. RTI stands for number of respiratory tract infections experienced over the first year of life.

**Fig. 3:**
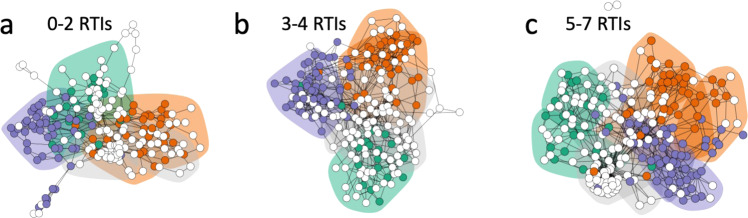
Cross-niche networks for each RTI group at week 1. Microbial cross-niche networks using data from the nasopharynx, gut, and oral cavity (**a**–**c**). Nodes represent individual OTUs and were coloured depending on their indicator species identity (orange = faeces-specific, green = nasopharynx-specific, blue = saliva specific, white = not niche-specific). Shaded areas around groups of nodes represent clusters defined by walktrap community analysis and are shaded depending on their niche identity (orange = faeces-specific, green = nasopharynx-specific, blue = saliva specific, grey = mixed cluster/not niche-specific).

We found that at 1 week of age, the network of the least susceptible group (0–2 RTIs) was structured into six clusters (one indicative of nasopharynx, one saliva, two faeces, and two mixed), while the network of the average susceptible group (3–4 RTIs) contained seven clusters (one nasopharynx, one saliva, two faeces, and three mixed) and the network of the most susceptible group (5–7 RTIs) contained nine clusters (two nasopharynx, two saliva, three faeces, and two mixed; Supplementary Fig. 3). The number of clusters per network generally decreased over time with cluster sizes increasing (Fig. 2a, b). However, cluster sizes in the networks from the 5–7 RTI group showed a trend towards less increase over time compared to clusters from networks from the 0–2 RTI group (negative binomial GLM: 5–7 RTI group estimate = − 0.3, *p* = 0.071), which coincided with a higher number of clusters retained in the 5–7 RTI networks. This resulted in the networks of the lowest and average susceptible RTI groups each defined by four clusters each, whereas the network from the most susceptible group still contained six clusters at the age of 6 months, suggesting more fragmented networks. The two extra clusters identified in the most susceptible group had a saliva and faecal origin, thereby split the oral and gut communities into two communities each (Supplementary Fig. 3). Figure 3 depicts the networks of each RTI group at time point week 1.

**Fig. 4:**
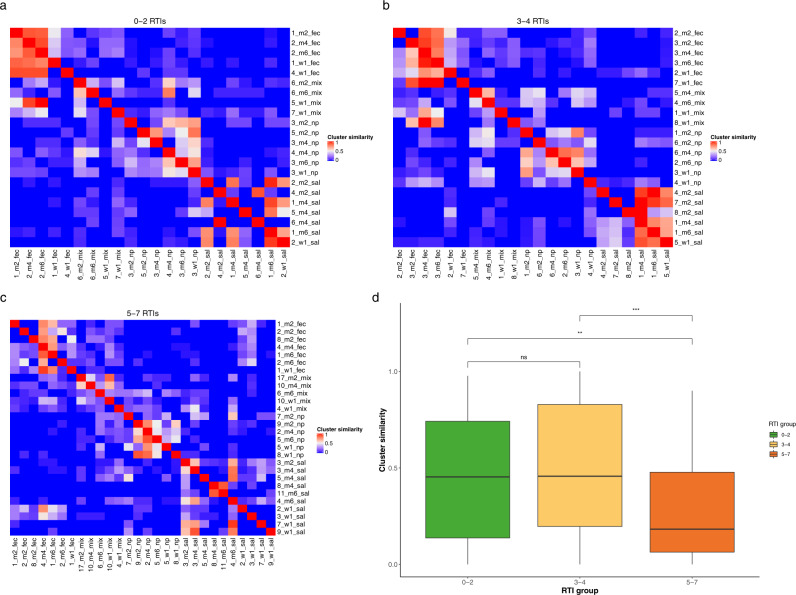
Stability and similarity of individual network clusters. Heatmaps (**a**–**c**) displaying the similarity between the cluster compositions of all clusters at all time points for networks for the 0–2 RTI group, 3–4 RTI group and 5–7 RTI group, respectively. Cluster similarity was calculated as the proportion of OTUs shared between two clusters (1 represents complete overlap between all OTUs of two clusters, whereas 0 represents no OTUs being shared between two clusters). Each cluster is labelled with a unique number to distinguish between clusters with the same niche-identity, the time point of the network from which the cluster originates (w1 = week 1, m2 = month 2, m4 = month 4, m6 = month 6) and the niche-identity of the cluster (fec = faecal cluster, np = nasopharyngeal cluster, sal = saliva cluster, mix = no clear niche-identity of cluster). **d** Mean cluster similarity for all clusters per RTI group across time. The number of clusters that are compared to each other is *n* = 22 for 0–2 RTIs, *n* = 22 for 3–4 RTIs, and *n* = 28 for 5–7 RTIs. Boxplots with medians are shown; the lower and upper hinges correspond to the first and third quartiles (the 25th and 75th percentiles); the upper and lower whiskers extend from the hinge to the largest and smallest value no further than 1.5 *IQR from the hinge. In order to assess the extent by which microbial communities from multiple body sites are linked and related to disease susceptibility, Reyman and Clerc et al. combined 16S-based rRNA sequencing data from 112 healthy, term born infants, spanning three body sites (oral cavity, nasopharynx, and gut) over the first 6  months of life. They demonstrate a strong association between network structure and species and susceptibility to respiratory tract infections, suggesting a crucial role of cross-niche microbial connections in respiratory health.

We then studied the similarity of clusters over time as a measure of temporal network stability (Fig. 4). The mean similarity over time differed between RTI groups, with 45.6% ± 4.9 SE similarity over time for the least susceptible group, 51.8% ± 5.1 SE for the average susceptible group and 29.0% ± 3.2 SE for the most susceptible group (Kruskal–Wallis test: X2 = 15.7, *p* = 0.0004, Fig. 4d). Specifically, the similarity of clusters in the networks of the most susceptible group was lower compared to the other two RTI groups (Dunn post-hoc test, 0–2 vs 3–4 RTIs: *p* = 0.328, 0–2 vs 5–7 RTIs: *p* = 0.012, 3–4 vs 5–7 RTIs: *p* = 0.0006; Fig. 4d). In stratified analyses per niche, we found significant differences in cluster similarity between RTI groups for saliva clusters (similarity over time 44.0% ± 9.9 SE in the 0–2 RTI group, 78.9% ± 6.7 SE in the 3–4 RTI group, and 19.6% ± 4.5 SE in the 5–7 RTI group, Kruskal–Wallis test X2 = 19.1, *p* < 0.0001, Supplementary Fig. 4). In addition, we found non-significant trends for the association between RTI susceptibility and loss in cluster similarity for faecal clusters (similarity over time 49.9% ± 11.1 SE in the 0–2 RTI group, 49.4% ± 9.3 SE in the 3–4 RTI group, and 41.0% ± 6.9 SE in the 5–7 RTI group, Kruskal–Wallis test *X*^2^ = 1.00, *p* = 0.612, Supplementary Fig. 4) and nasopharyngeal clusters (similarity over time 54.5% ± 5.8 SE in the 0–2 RTI group, 42.7 % ± 7.4 SE in the 3-4 RTI group, and 35.3% ± 6.2 SE in the 5–7 RTI group, Kruskal–Wallis test X^2^ = 4.28, *p* = 0.118). Overall, this suggests reduced cluster stability over time in networks from children from the 5–7 RTI group.

### Hub species

To identify OTUs with an important role for the network structure, we calculated the degree, betweenness centrality and closeness centrality for each of the OTUs in each network (only considering main clusters). Table 1 lists the 134 hub species found, also reporting whether they were niche indicator species or not, and for which RTI network (least, average, or most susceptible) they were identified as being important. We identified only 5 OTUs (*Akkermansia* (280), *Moraxella* (190), *Actinomyces* (58), *Veillonella* (10) and *Prevotella melaninogenica* (22)) as hub species in all three RTI groups, indicating that different OTUs are central to the network structure of the different RTI groups.

**Table 1:** Hub species for all RTI cohorts.

OTU	Hub ID	Niche indicator	No. thresholds met
Actinomyces_58	All	Sal	1
Akkermansia_280	All	None	1
Moraxella_190	All	None	1
Prevotella_melaninogenica_22	All	Sal	1
Veillonella_10	All	Sal	1
7B_8_687	0–2	None	2
Acinetobacter_calcoaceticus_146	0–2	None	1
Aggregatibacter_354	0–2	None	1
Alloscardovia_316	0–2	None	2
Altererythrobacter_748	0–2	None	1
Anaerostipes_142	0–2	None	1
Blastococcus_281	0–2	None	2
Blastococcus_707	0–2	None	1
Blautia_212	0–2	None	1
Chroococcidiopsis_338	0–2	None	1
Chroococcidiopsis_593	0–2	None	3
Chryseobacterium_299	0–2	None	1
Corynebacterium_111	0–2	None	2
Corynebacterium_162	0–2	None	1
Craurococcus_sp_HM28_1_726	0–2	None	1
Cupriavidus_31	0–2	None	1
Curvibacter_286	0–2	None	1
Dorea_86	0–2	None	1
Erysipelotrichaceae_149	0–2	None	2
Lachnospiraceae_176	0–2	None	2
Limnobacter_245	0–2	None	2
Megasphaera_133	0–2	None	2
Megasphaera_sp_TrE9262_383	0–2	None	1
Moraxella_131	0–2	None	1
Neisseria_lactamica_47	0–2	None	1
Pseudobutyrivibrio_195	0–2	None	2
Rubellimicrobium_596	0–2	None	1
Ruminococcus_bromii_L2_63_112	0–2	None	2
Sphingomonas_573	0–2	None	1
Subdoligranulum_71	0–2	None	1
Varibaculum_199	0–2	None	1
Escherichia_Shigella_267	0–2	Fec	1
Comamonadaceae_118	0–2	Np	1
Dolosigranulum_pigrum_ATCC_51524_147	0–2	Np	1
Enhydrobacter_138	0–2	Np	1
Pseudomonas_158	0–2	Np	1
Haemophilus_211	0–2	Sal	1
Helcococcus_179	0–2 and 3–4	None	2
Scardovia_wiggsiae_F0424_127	0–2 and 3–4	None	2
Bifidobacterium_2	0–2 and 3–4	Fec	2
Rothia_259	0–2 and 3–4	Sal	1
Streptococcus_1	0–2 and 3–4	Sal	1
Actinomyces_100	0–2 and 5–7	None	1
Blautia_308	0–2 and 5–7	None	1
Moryella_119	0–2 and 5–7	None	1
Gallibacterium_Salpingitidis_141	0–2 and 5–7	Sal	1
Akkermansia_210	3–4	None	1
Akkermansia_40	3–4	None	1
boneC3G7_70	3–4	None	2
Clostridium_colinum_487	3–4	None	1
Clostridium_nexile_166	3–4	None	2
Corynebacterium_500	3–4	None	2
Corynebacterium_aurimucosum_ATCC_700975_260	3–4	None	2
Haemophilus_226	3–4	None	1
Lachnoanaerobaculum_115	3–4	None	1
Lactobacillus_plantarum_337	3–4	None	1
Modestobacter_323	3–4	None	1
Moraxella_204	3–4	None	1
Morganella_294	3–4	None	1
Neisseria_345	3–4	None	2
Peptococcus_like_sp_oral_clone_I070_371	3–4	None	2
Propionibacterium_312	3–4	None	2
Sphingomonas_228	3–4	None	1
Turicella_264	3–4	None	2
Zymomonas_335	3–4	None	2
Bifidobacteriaceae_213	3–4	Fec	1
Clostridium_sensu_stricto_1_33	3–4	Fec	1
Collinsella_220	3–4	Fec	1
Eggerthella_189	3–4	Fec	2
Klebsiella_13	3–4	Fec	1
Corynebacterium_350	3–4	Np	1
Moraxella_3	3–4	Np	1
Alloprevotella_23	3–4	Sal	1
Bergeyella_108	3–4	Sal	1
Lachnoanaerobaculum_128	3–4	Sal	1
Lactobacillales_63	3–4	Sal	1
Rothia_126	3–4	Sal	1
Rothia_16	3–4	Sal	1
Rothia_192	3–4	Sal	1
Rothia_287	3–4	Sal	1
Solobacterium_moorei_232	3–4	Sal	1
Streptococcus_20	3–4	Sal	1
Veillonella_66	3–4	Sal	1
Collinsella_110	3–4 and 5–7	None	1
Lachnospiraceae_98	3–4 and 5–7	None	2
Campylobacter_123	3–4 and 5–7	Sal	1
Veillonella_sp_DNF00869_15	3–4 and 5–7	Sal	1
Actinobaculum_schaalii_FB123_CNA_2_408	5–7	None	1
Bacteroides_84	5–7	None	2
Capnocytophaga_196	5–7	None	2
Erysipelotrichaceae_102	5–7	None	1
Erysipelotrichaceae_231	5–7	None	2
Flavonifractor_200	5–7	None	1
Fusicatenibacter_saccharivorans_44	5–7	None	1
Haemophilus_haemolyticus_663	5–7	None	1
Lachnospiraceae_144	5–7	None	2
Lachnospiraceae_230	5–7	None	2
Lachnospiraceae_302	5–7	None	1
Lactobacillus_fermentum_91	5–7	None	1
Moraxella_153	5–7	None	1
Moraxella_412	5–7	None	1
Moraxella_494	5–7	None	1
Neisseria_1000	5–7	None	1
Neisseria_469	5–7	None	2
Neisseria_527	5–7	None	1
Neisseria_949	5–7	None	1
Neisseria_meningitidis_830	5–7	None	1
Parascardovia_denticolens_F0305_486	5–7	None	1
Peptoniphilus_sp_S7MS8_148	5–7	None	1
Peptostreptococcaceae_117	5–7	None	1
Peptostreptococcaceae_75	5–7	None	1
Prevotella_sp_oral_clone_ID019_94	5–7	None	1
Ruminococcaceae_160	5–7	None	2
Ruminococcaceae_358	5–7	None	1
Streptococcus_Salivarius_subsp_thermophilus_364	5–7	None	1
Subdoligranulum_249	5–7	None	1
Bacteroides_74	5–7	Fec	1
Bifidobacterium_dentium_Bd1_28	5–7	Fec	1
Blautia_19	5–7	Fec	1
Collinsella_26	5–7	Fec	1
Erysipelotrichaceae_78	5–7	Fec	1
Parabacteroides_distasonis_82	5–7	Fec	1
ratAN060301C_27	5–7	Fec	1
Ruminococcus_gnavus_CC55_001C_24	5–7	Fec	1
Haemophilus_8	5–7	Np	1
Actinomyces_sp_oral_clone_DR002_81	5–7	Sal	1
Haemophilus_215	5–7	Sal	1
Haemophilus_413	5–7	Sal	1
Veillonella_156	5–7	Sal	2

Np nasopharynx, sal saliva, Fec faeces, None no niche indicator OTU.

The column niche indicator shows whether an OTU is also a niche indicator OTU. Hub ID indicates for which RTI group (or combination of RTI groups) an OTU is a hub species. Rows are ordered firstly by hub ID, then niche indicator (None, faeces, np or saliva), then on OTU in alphabetical order.

To assess whether the identified hub species were high or low abundant species, we calculated summary statistics for all hub species split by the RTI group(s) for which they were identified as hub species (Supplementary Table 3). The mean abundance of hub species in the 0–2 RTI group networks was 0.0150% ± 0.54 SD (range 0–55.6%), 0.34% ± 3.8 SD for the 3–4 RTI group (range 0–99.5%) and 0.12% ± 1.8 SD (range 0–96.5%) for the 5–7 RTI group underlining hub OTUs are often low abundant species. Furthermore, most hub species were not identified as niche indicator OTUs. This suggests that niche indicator OTUs are less relevant for the microbial network structure and vice versa, that hub species are less niche-specific.

The hub species unique to the 0–2 RTI networks included previously reported respiratory health-associated OTUs such as *Neisseria lactamica* (47), a low abundant lactic acid-producing *Dolosigranulum pigrum* (147), and *Corynebacterium* (162 and 111) OTUs. Also, OTUs associated with the production of butyrates, such as *Ruminococcus bromii* (112), *Megasphaera* (133 and 383), and *Anaerostipes* (142), were found to be hub species in the 0–2 RTI networks. Alternatively, hub species in the 5–7 RTI networks included OTUs previously associated with poorer respiratory health, such as the proteobacterial OTUs *Haemophilus* (8, 215, 413), *Haemophilus haemolyticus* (663), and *Neisseria* (469, 527, 949, 1,000), and the anaerobic *Lachnospiraceae* (144, 230, 302). Additionally, *Actinobaculum schaalii* (408) is often associated with (invasive) infections, the proinflammatory *Ruminococcus gnavus* (24) and the faecal pH increasing *Peptostreptococcaceae* (75 and 117) were hub species for the networks of infants who experienced 5–7 RTIs. Also, cariogenic species such as *Bifidobacterium dentium* (28) and *Parascardovia denticolens* (486) were identified as hub species in the 5–7 RTI networks.

## Discussion

Previous studies from this healthy Dutch birth cohort showed already that the nasopharyngeal, oral, and gut microbiota individually develops along specific trajectories, and that various environmental factors are associated with the microbial community composition and development of these three niches in the first months of life. Also, associations between the microbiome development of each of these separate niches and respiratory health have been observed, with an accelerated maturation of the nasopharyngeal microbiome being associated with the number of RTIs experienced in the first year of life, a loss of topography of the upper respiratory microbiome (oropharynx and nasopharynx) being shown to precede RTIs and an association is found between the faecal microbiota composition at one week of life with the number of RTIs experienced in the first year of life^10–12^. Although this suggests an interplay between the microbial communities across these niches, this has previously not been studied. Here, we investigated cross-niche infant microbiota composition and development and its relationship with susceptibility to RTIs within the first year of life. We show multiple pieces of evidence that support a link between network structure and RTI susceptibility, which are (1) the increase in network fragmentation (increased number of clusters) with increase in RTI susceptibility, (2) the decrease in network stability (decrease in cluster similarity) with an increased RTI susceptibility, and (3) the identification of hub species associated with respiratory health in the least susceptible RTI group vs. identification of hub species associated with (respiratory) dysbiosis in the most susceptible RTI group. To our knowledge, this is the first study using a cross-niche microbial network strategy to study the composition of the wider infant microbiome in relation to (respiratory) health.

In the rapidly developing early-life microbiome, we observed that the anatomical niche was by itself the most important explanatory variable for the microbial community composition, suggesting that the niche environment is the main driver of the composition of the local microbiome. This has already been found in other studies^8^, but we also identified additional environmental drivers of microbiota composition and development, among others mode of delivery, feeding type and the presence of siblings <5 years in the household^11–13^.

Building cross-niche microbial networks allowed us to additionally investigate the connections between local and distal microbial communities across the body and how this related to respiratory health. In doing so, we were not only able to identify clusters of OTUs in the cross-niche networks that were niche-specific but also clusters of bacteria that were niche-independent. The network clusters found to be dominated by niche-specific bacteria are in line with previous results from human microbiome network studies^8^.

When analysing the cross-niche network structure in relation to RTI susceptibility, we observed that the number of clusters within a network was highest for the most susceptible group, while the temporal stability of clusters was lowest. This demonstrates that increased susceptibility to RTIs might (in part) be driven by an increase in network fragmentation, which is further characterised by a decrease in similarity across clusters with the same niche-specificity. Of note is the fact that we were unable to statistically test the difference in cluster numbers across time and RTI group, as for each of these categories, only one network could be built given the available data. However, our finding of fragmentation is in line with a study focusing on the structure of (niche-specific) gut microbial co-occurrence networks in IBD patients, which was more distorted when compared to networks in healthy individuals^7^. Further, a theoretical study by Ma et al. supports our observation by showing that properties of critical network structures were associated with microbiome-associated diseases^14^.

When studying hub OTUs central to the respective cross-niche networks^15,16^, we observed little overlap between RTI groups. In order to place the identity of the hub OTUs that we identified into a disease/health-related context, we performed a non-systematic literature search for associations between hub species and (respiratory) health. We found OTUs previously associated with low susceptibility to, and low severity of, respiratory infections, such as *Corynebacterium, D. pigrum* and *N. lactamica*, as hub species in networks from our least susceptible group. Both *Corynebacterium* and *D. pigrum* have been consistently associated with a health-associated microbiota maturation and a decreased risk of developing RTIs in later life^11,17,18^. Furthermore, *N. lactamica* has been shown in human challenge studies to inhibit colonization by the pathogenic *Neisseria meningitidis*, which supports that *N. lactamica* plays a role in a resilient microbial community network^19^. Inversely, we observed hub species that were previously associated with recurrent respiratory infections, such as *Haemophilus* and *Lachnospiraceae*, in the networks of the group experiencing the most RTIs, but generally not in the group with few RTI episodes^11,20–22^. Therefore, our data suggest that the presence of these bacteria is not incidental but potentially central to a less beneficial bacterial community structure associated with more RTI episodes.

Not only did we identify hub species that were previously associated with respiratory health, but we also identified OTUs that were previously associated with gut health, oral health, immunological diseases, and infections as hub species in the networks of infants experiencing either 0–2 or 5–7 RTIs. With respect to gut health, beneficial OTUs known to produce or enhance the production of butyrates, such as *R. bromii* (112), *Megasphaera* (133 and 383), and *Anaerostipes* (142), were found to be hub species in the networks of the least susceptible group^23–25^. The short-chain fatty acid butyrate is a microbial end-product of the human gut fermentation process and an essential metabolite in the gut environment, being the preferred energy source for colon epithelial cells. It has anti-inflammatory properties and lowers the pH of the gut environment, in this way inhibiting growth of pathogens^23^. Conversely, *Peptostreptococcaceae* spp. (75 and 117), previously associated with increased faecal pH, were identified as hub species for the networks from the most susceptible group^26^. Additionally, in this group, the mucin degrader *R. gnavus* (24) was identified as a hub species. This OTU has previously been associated with a broad range of immunological disorders, such as paediatric allergy, IBD and psoriatic arthritis, and also with failure of faecal microbiota transplantation^27–32^. Also, in the most susceptible RTI networks, we observed *A. schaalii* (408), involved in urinary tract infections, as hub species, as well as the cariogenic *B. dentium* (28) and *P. denticolens* (486)^33–35^. Altogether, these observations suggest that cross-niche bacterial networks may stand at the basis of overall systemic susceptibility to inflammation-driven health conditions. They further imply that the overall construction of microbial networks, in addition to merely the presence/absence or abundance of specific commensals or pathogens, can act as a driving force behind inflammation-mediated disease.

Of note, although the more predominant OTUs were generally niche-specific, most hub species were actually lower abundant taxa and not associated with a specific niche. This supports the “rare taxa” concept, which postulates that the abundance of a species is not necessarily the best determinant for its importance within the microbial community structure^36^. Studying the human microbiome in a more system-centric context, therefore, might provide insight into the importance and roles of lesser-known microbes. In addition, it is important to highlight the fact that we were able to shed some light on the degree of cross-niche connectivity between microbial communities within the body.

Relationships between microbial communities across body sites have already been pointed out, for example, by Madan et al.^37^, who showed that the introduction of solid foods in children with cystic fibrosis not only changed the microbiome composition within the gut but also that of the respiratory tract. These connections are likely a result of indirectly connected microbe-host interactions, such as immunological pathways triggered by one microbe that affect distally located microbes or metabolic products used by distally located microbes^38,39^, rather than direct microbe–microbe interactions, which is due to their physical separation, less likely. Examples for such effects of distally located microbes affecting susceptibility to RTI’s have already been presented in the literature. Using a gain-of-function genetic mouse model, Steed et al. were able to show that desaminotyrosine produced by the human gut commensal *Clostridium orbiscindens* augmented IFN-I signalling, thereby rendering protection from influenza morbidity and mortality in infected mice^40^. In addition, Olsen and Yamazaki present a mechanism by which systemic diffusion of inflammatory mediators from periodontal lesions can lead to or contribute to the development of a multitude of gastrointestinal conditions such as non-fatty liver disease, rheumatoid arthritis, or cancer^41^.

One key strength of our study is the integrated use of microbiota data across different body sites to build cross-niche microbial networks in a longitudinal fashion, using samples collected from a prospectively followed birth cohort of children recruited at a single hospital in the Netherlands. By using a cross-niche network approach with an algorithm based on conditional independence rather than correlation, we showed that besides the known associations between niche-specific microbiota and respiratory health, there is likely also an association between the overall infant microbiome network features and respiratory health. Furthermore, we were able to identify hub species that were important for the network structure, which were often niche-independent, suggesting these microbes might be missed or their value might be underestimated when the microbiota is studied within a single niche. With respect to future studies, our results demonstrate that careful consideration of the composition and connectivity of local and distal microbial communities can enhance our understanding of the magnitude of systemic dysbiosis potentially underlying health and disease. However, we are aware that experimental limitations might not always allow the study of the composition of the infant microbiome on a systemic scale, although studies like ours may still provide a framework to formulate testable hypotheses that incorporate more than one niche community.

Limitations of our study are mainly inherent to the experimental design, including only children recruited at a single hospital in the Netherlands, warranting the need for similar studies covering a broader range of geographical variation. In addition, future in vitro/in vivo studies are needed to understand the biological mechanisms underpinning these findings. Furthermore, we potentially lack statistical power when comparing network cluster similarities across RTI groups within niches, as there were only between one and four niche-specific clusters in any one network. Lastly, we only considered univariate relationships between environmental factors and infant microbiome composition as a means to pre-select factors to be combined in a multivariate model. This approach allowed us to pre-screen a large number of covariates for their potential effect on the infant microbiome without the risk of overfitting. However, it does not take into account potential interactions/correlations between individual covariates.

In conclusion, we observed cohesive and stable microbial networks across body sites already from early life on, which in turn were associated with a lower susceptibility for RTIs in the first year of life. In contrast, we found more fragmented and unstable cross-niche networks over the first 6 months of life in infants with higher susceptibility to RTIs. Finally, we identified bacteria that were central to each of the cross-niche microbial network structures, though these differed between RTI groups. These bacteria were often not indicative for a specific niche within the body, and represented low abundant species, underlining the potential importance of low abundant bacteria for microbiome function.

## Methods

### Data collection

A detailed description of the study design and inclusion criteria of the birth cohort can be found elsewhere^13^. In short, we used microbiota data from samples obtained from 112 healthy infants born at term (≥37 weeks gestational age) recruited at a single Dutch hospital at four different time points (week 1, month 2, month 4, and month 6) and from three different niches, namely the gastrointestinal tract (faecal samples), the upper respiratory tract (nasopharyngeal swabs), and the oral cavity (saliva samples). Metadata was available for the first year of life, but matched samples collected in parallel were only available until month 6. Written informed consent was obtained from parents of all children and the study was approved by METC Noord-Holland (M012-015, NTR3986). Infants were divided into three groups based on the distribution of the number of RTIs they experienced over the first year of life (0–2 RTIs, 3–4 RTIs, 5–7 RTIs), and as described in previous publications using data from this birth cohort Supplementary Fig. 5)^10^.

### 16S rRNA gene sequencing

DNA extraction and library preparation of the bacterial 16S rRNA gene V4 region was performed as described previously^13^. Samples were sequenced in 27 individual library pools, each containing DNA extraction and qPCR blanks as negative control; and a mock community (positive control). All pools were sequenced on the Illumina MiSeq platform (Illumina Inc., San Diego, CA, USA). The raw sequencing reads were trimmed using Sickle v1.33 (with the quality threshold set to *q* = 30 and the length threshold set to 150 nucleotides)^42^; error corrected using BayesHammer (SPAdes v3.8.1)^43^ and assembled using PANDAseq v2.10^44^. Following removal of chimeric sequences, reads were grouped into Operational Taxonomic Units (OTUs) using VSEARCH v2.0.3 with a 97% similarity threshold^45^. Taxonomic annotation was performed using QIIME v1.9.1 based on the SILVA database v119^46,47^. We removed spurious OTUs by filtering at a minimum relative abundance of 0.1% and presence in at least two samples^48^. We combined each OTU name with a number representing its rank in the OTU table, based on the relative abundance in the overarching dataset to discriminate OTUs with the same taxonomic annotation.

### Statistical analysis

Analyses regarding the microbiota composition have been performed in R version 3.4.3, while network analyses have been performed in R versions and 3.6.2^49^.

### Niche comparison

Results of analyses of observed species richness were based on raw read counts (Supplementary Fig. 6 for rarefaction curves). Similar results were obtained when rarefying read counts to a sequencing depth of 3,000 reads. We used the lmer function (lme4 package)^50^ to study the temporal changes of species richness within and across niches, with participants added as random effect to take repeated measures into account. To compare compositions of microbial communities across niches, we used non-metric multidimensional scaling (nMDS) plots based on ordinations that used the Bray–Curtis (BC) dissimilarity matrix of relative abundance data as input (function *ordinate* with parameter trymax 10,000; vegan package)^51^. Stability of the microbiota composition in the first 6 months of life within each niche was visualised by measuring the BC dissimilarities between consecutive samples of each participant, and we tested differences across niches using Wilcoxon’s signed rank test.

We used permutational multivariate analysis of variance (PERMANOVA) with 1,999 permutations to investigate the associations between microbiota composition (response variable) and environmental covariates using the function *adonis2* (vegan package)^51^. The covariates tested were mode of delivery (vaginal vs. caesarean section), season of birth (summer, autumn, winter, spring), hospital stay duration after birth in day parts, presence of siblings <5 years of age (yes vs. no), presence of pets in the household (none, cat(s), dog(s), cat(s) and dog(s), other), breastfeeding at sampling moment (yes vs. no), attendance of day care at sampling moment (yes vs. no), use of pacifier at sampling moment (yes vs. no) and use of antibiotics in the month prior to sampling moment (yes vs. no). Covariates that were significantly associated in univariate models (per time point per niche, Supplementary Figs. 7–12) with microbiota community composition at a minimum of one-time point were included in a multivariate model that included niche as well as age and subject to control for repeated measures. *P*-values were adjusted for multiple testing using the Benjamini–Hochberg method^52^.

### Network construction and cluster definition

Prior to network construction, we split the data by RTI group and filtered the OTU tables to only include the 100 most abundant OTUs per niche, and within each RTI group in order to avoid overestimation of the impact of very rare taxa on the overall network structure. Because of overlapping OTUs between niches, this resulted in a final dataset that included 220 OTUs for the 0–2 RTI group, 214 OTUs for the 3–4 RTI group and 228 OTUs for the 5–7 RTI group. All combined, we identified 315 non-redundant OTUs, of which 149 OTUs were present in all three groups (Supplementary Fig. 13). Using the top 100 prevalent OTUs per niche instead of the 100 most abundant OTUs resulted in a smaller dataset of only 251 OTUs in total. Seventy-four percent of those 251 OTUs identified using a prevalence filter were also identified using an abundance filter, and since the overlap between the two methods was large, we decided to continue our analysis using the abundance filter since this resulted in a bigger number of OTUs to be available for network construction.

We defined niche indicator OTUs as OTUs that were characteristic for a particular niche. To identify such OTUs, we ran a niche indicator species analysis using the function *multipatt* (indicspec package)^53^. This function calculates an indicator value for each OTU at each niche, taking its total abundance per niche into account. An OTU has deemed a niche indicator when it had an association value of >0.5 with a *p*-value < 0.05 for one specific niche.

For network construction, we used the SpiecEasi pipeline with Meinshausen–Buhlmann estimation (number of StARS repetitions = 50, lambda scaling factor = 1e-2)^9^. This method is based on the concept of conditional independence rather than correlation, making it less likely to detect spurious connections between taxa that are indirectly connected but not directly connected. Further, it uses centred log-ratio transformation of the data to overcome the compositionality of microbiome data. In total, we constructed 12 networks (3 RTI groups × 4 time points) using the methods described above, with the number of OTUs remaining constant in each network. We used random walks (number of steps = 5) to group OTUs into clusters that were closely associated using the function *walktrap.community* (igraph package)^54^. We chose a step length of five since the number of clusters plateaued after this value (Supplementary Fig. 14). We considered clusters with ten or more OTUs as “main clusters”, as smaller clusters usually contained only one or two OTUs (Supplementary Fig. 15). The OTUs within a cluster can be either niche indicator OTUs (saliva, faeces, or nasopharynx) or non-niche indicator OTUs. Therefore, if the composition of a cluster were random, we would expect an equal proportion of any of these four OTU classes within a cluster, i.e. each OTU class would have a prevalence of 25%. We considered a cluster niche-specific if one of the OTU classes exceeded that threshold value of 25%. This is a very conservative threshold as none of the classes of niche indicator OTUs actually made up 25% of OTUs (between 5.7% and 11.5% of OTUs within an RTI group). The use of this conservative threshold allowed us to unambiguously assign cluster identity. If there were two classes of niche indicator OTUs that met this threshold within a single cluster, then the largest set determined the niche annotation. If there were not enough niche indicator OTUs that met this threshold, the cluster was defined as a “mixed (niche) cluster”.

### Cluster stability analysis

We compared the composition of all clusters from each time-specific network per RTI group using an n × n matrix, where cluster size was not taken into account. We then calculated the difference in cluster composition from a scale from 0 to 1, where 1 indicates complete similarity (i.e., two clusters share 100% of their OTUs). We used ANOVA to assess whether cluster similarity was different across niches.

### Hub species analysis

To identify hub species (i.e., OTUs playing a significant role in determining the structure of a community network), we calculated three important network metrics for each OTU within a network at each time point: (1) degree (the number of connections each OTU has within a network), (2) betweenness centrality (number of shortest paths that pass through a specific OTU within a network), and (3) closeness centrality (the reciprocal of the sum of the length of the shortest paths between an OTU and all other OTUs in a network). The correlation data between network metrics is shown in Supplementary Fig. 16. We followed the definitions from Banerjee et al. and Berry and Widder by defining hub species as those OTUs with a high degree, high closeness centrality and low betweenness centrality^15,16^. We defined high degree or closeness centrality as the top 10% of the distribution of each of those metrics and low betweenness centrality as the bottom 10% of the distribution for betweenness. By doing so, we ensured a selection of OTUs that were at the extremes of the distributions for all three values (see Supplementary Fig. 17). We selected OTUs within RTI groups that met at least one of those conditions as hub species.

### Reporting summary

Further information on research design is available in the Nature Research Reporting Summary linked to this article.

## Supplementary information

Supplementary information

Description of Additional Supplementary Files

Supplementary Data 1

Reporting Summary

## Data Availability

Raw sequence data underlying the findings of this study have been previously deposited in the NCBI Sequence Read Archive (SRA) database as part of other publications with BioProject IDs PRJNA353336, PRJNA450937, and PRJNA481243. The results presented in this manuscript are based on a subset of the sequences deposited under these BioProjects. The source data underlying the tables and figures presented in this manuscript are provided in Supplementary Data 1.
