# Supplementary material for: Microbial community networks across body sites are associated with susceptibility to respiratory infections in infants
Source: Commun Biol. 2021 Oct 28;4:1233. doi: 10.1038/s42003-021-02755-1 (PMC8553847; doi:10.1038/s42003-021-02755-1)

Supplementary Information

Microbial community networks across body sites are associated with susceptibility to respiratory infections in infants

Marta Reyman[1,2,3*], Melanie Clerc[4,6*], Marlies A. van Houten[2], Kayleigh Arp[1,5], Mei Ling J.N. Chu[1,5], Raiza Hasrat[1,5], Elisabeth A.M. Sanders[1,5], Debby Bogaert[1,4]

1 Department of Paediatric Immunology and Infectious Diseases, Wilhelmina Children's Hospital of University Medical Centre, Utrecht, The Netherlands

2 Spaarne Gasthuis Academy Hoofddorp and Haarlem, the Netherlands

3 Department of Dermatology, Erasmus Medical Center, Rotterdam, the Netherlands (current address)

4 University of Edinburgh Centre for Inflammation Research, Queen's Medical Research Institute, University of Edinburgh, United Kingdom

5 National Institute for Public Health and the Environment, Bilthoven, the Netherlands

6 Institute of Microbiology, ETH Zürich, Zürich, Switzerland (current address)

* Both authors contributed equally to this manuscript

Correspondence to:

Prof Debby Bogaert, Medical Research Council and University of Edinburgh Centre for Inflammation Research, Queen's Medical Research Institute, University of Edinburgh, Edinburgh EH16 4TJ, UK

d.bogaert@ed.ac.uk

Supplementary Tables

Supplementary Table 1: Results of multivariate, longitudinal PERMANOVA analysis. We performed permutational multivariate analysis of variance (PERMANOVA) with 1999 permutations to investigate the associations between microbiota composition (response variable) and environmental covariates. Covariates that were significantly associated in univariate models (per time point per niche) with microbiota community composition at a minimum of 1 time point were included in a multivariate model that included niche as well as age, and subject to control for repeated measures. The effect sizes (R^2) and adjusted p-values (using Benjamini-Hochberg method to correct for multiple testing) are shown.

**Covariate**	**R^2 (%)**	**adjusted *p***
Niche	42.4	0.00079
Age	1.8	0.00079
Siblings <5 years present	0.3	0.00079
Pets	0.3	0.00079
Breastfeeding at sampling	0.3	0.00079
Season of birth	0.2	0.00079
Mode of delivery	0.2	0.00079
Antibiotics 1 month prior to sampling	0.1	0.041
Day care attendance	0.1	0.041
Pacifier use	0.1	0.041
Hospital duration postpartum	0.1	0.019

Supplementary Table 2: Results of univariate, cross-sectional PERMANOVA analyses performed per niche. We performed permutational multivariate analysis of variance (PERMANOVA) with 1999 permutations to investigate the associations between microbiota composition (response variable) and environmental covariates. The effect sizes (R^2), p-values, adjusted p-values (using Benjamini-Hochberg method to correct for multiple testing), and p-values to test for overdispersion (beta-disper *p*) are shown. Pp = postpartum, antibiotics 1 month prior = antibiotics administered in the month prior to sampling (for the 1 week time point this refers to antibiotics administered in the first week of life).

**Niche**	**Time point**	**Covariate**	**R^2 (%)**	***p***	**adjusted *p***	**beta-disper *p***
**Faeces**	Week 1	Mode of delivery	14.4	0.0005	0.0020	0.035
		Hospital duration pp	11.9	0.0005	0.0020	0.052
		Breastfeeding at sampling	2.7	0.0095	0.019	0.50
		Antibiotics 1 month prior	2.6	0.0055	0.015	0.99
	Month 2	Breastfeeding at sampling	5.0	0.001	0.009	0.064
		Siblings <5 years present	4.1	0.002	0.009	<0.001
		Mode of delivery	2.0	0.038	0.11	0.63
	Month 4	Day care attendance	3.4	0.007	0.036	<0.001
		Breastfeeding at sampling	2.9	0.008	0.036	0.22
		Siblings <5 years present	2.1	0.042	0.13	0.037
	Month 6	Siblings <5 years present	3.0	0.012	0.10	0.006
		Day care attendance	2.5	0.023	0.10	0.027
**Naso-pharynx**	Week 1	Pets	7.5	0.0085	0.023	0.22
		Mode of delivery	3.6	0.0080	0.023	0.51
		Hospital duration pp	3.4	0.0050	0.023	0.077
		Breastfeeding at sampling	2.9	0.015	0.029	0.11
		Siblings <5 years present	2.8	0.018	0.029	0.075
	Month 2	Siblings <5 years present	4.4	0.0010	0.0090	0.62
		Breastfeeding at sampling	2.5	0.024	0.071	0.049
		Antibiotics 1 month prior	1.8	0.013	0.056	0.001

	Month 4	Season of birth	4.7	0.050	0.150	0.40
		Siblings <5 years present	3.3	0.0065	0.059	0.011
		Pacifier use	2.5	0.024	0.11	0.34
	Month 6	Season of birth	5.2	0.022	0.066	0.42
		Siblings <5 years present	3.6	0.0015	0.014	0.021
		Day care attendance	2.9	0.0065	0.029	0.014
**Saliva**	Week 1	Antibiotics 1 month prior	3.9	0.0085	0.04	0.039
		Breastfeeding at sampling	3.1	0.010	0.04	0.13
	Month 2	Breastfeeding at sampling	5.9	0.0005	0.0045	0.077
	Month 4	Breastfeeding at sampling	7.3	0.0005	0.0045	0.074
		Season of birth	6.2	0.0075	0.034	0.15
	Month 6	Season of birth	6.0	0.011	0.038	0.088
		Antibiotics 1 month prior	3.8	0.013	0.038	0.22
		Breastfeeding at sampling	3.7	0.0045	0.038	0.94

Supplementary Table 3: Summary statistics of relative abundance of hub species split by RTI group specificity. Shown are mean relative abundance, standard deviation of relative abundance, median relative abundance, minimum relative abundance and maximum relative abundance.

RTI group specificity	mean relative abundance	SD relative abundance	median relative abundance	minimum relative abundance	maximum relative abundance
0-2	0.00015	0.00535	0	0	0.556
3-4	0.00342	0.0376	0	0	0.995
5-7	0.00124	0.0187	0	0	0.965
0-2 and 3-4	0.0743	0.204	0	0	0.995
0-2 and 5-7	0.000209	0.00459	0	0	0.296
3-4 and 5-7	0.00477	0.0245	0	0	0.398
all groups	0.00687	0.0282	0	0	0.508

Supplementary Figures

Supplementary Figure 1: Relative abundance of the most abundant OTUs. Stacked bar plots depicting mean relative abundance of the top 15 OTUs at each of 4 time points per niche (a = faeces, b = nasopharynx and c = saliva). The number in parentheses refers to the rank of an OTU in the OTU table and is based on its overall relative abundance in the dataset.

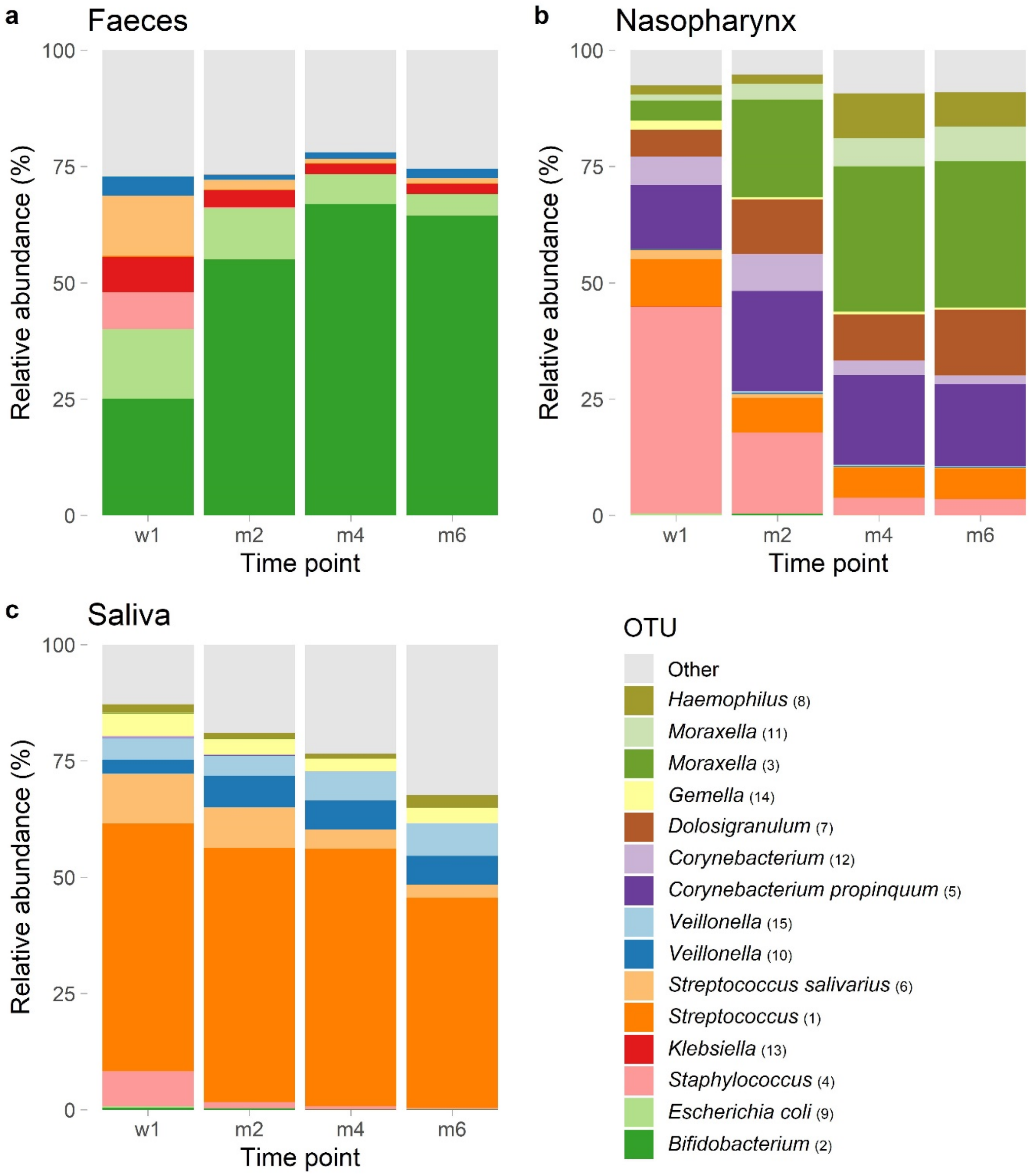

Supplementary Figure 2: Observed species richness. Observed species richness (number of species per sample) is shown per time point and niche. Boxplots with medians are shown; the lower and upper hinges correspond to the first and third quartiles (the 25th and 75th percentiles); the upper and lower whiskers extend from the hinge to the largest and smallest value no further than 1.5*IQR from the hinge; outliers are plotted individually by opaque circles; translucent circles visualise all data points.

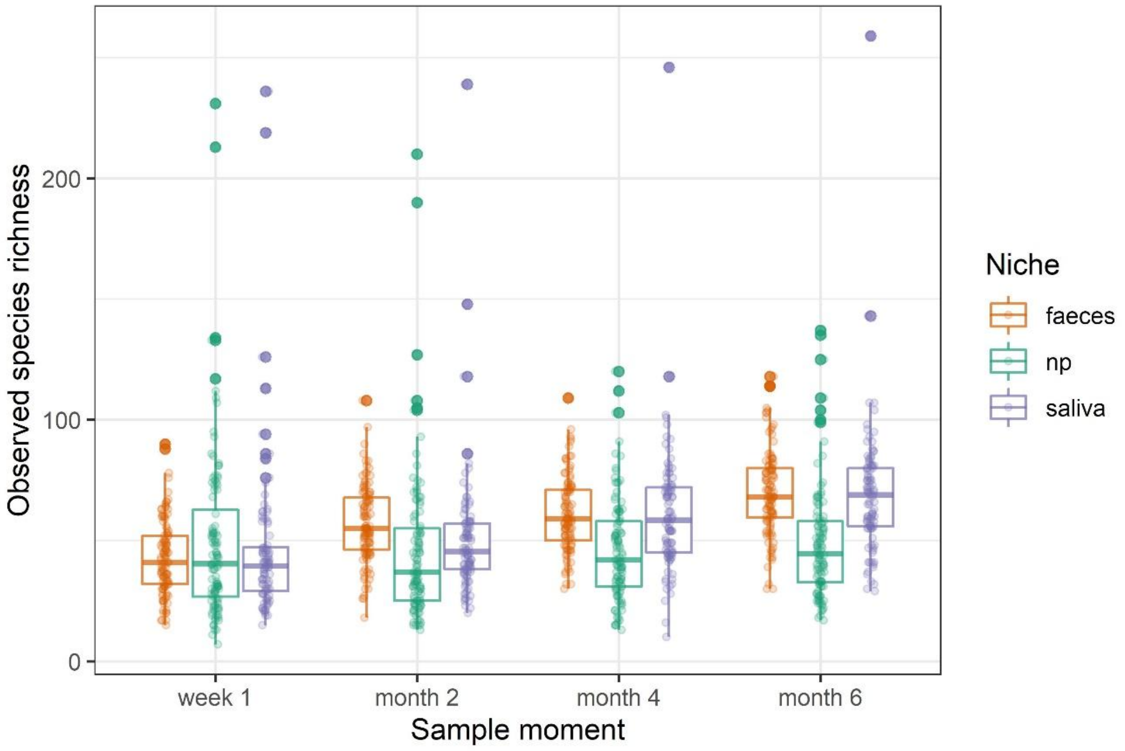

Supplementary Figure 3: Cross-niche networks for each RTI group per time point. Nodes represent individual OTUs and were coloured depending on their indicator species identity (orange = faeces-specific, green = nasopharynx-specific, blue = saliva specific, white = not niche-specific). Shaded areas around groups of nodes represent clusters defined by walktrap community analysis and are shaded depending on their niche-identity (orange = faeces-specific, green = nasopharynx-specific, blue = saliva specific, gray = mixed cluster/not niche-specific).

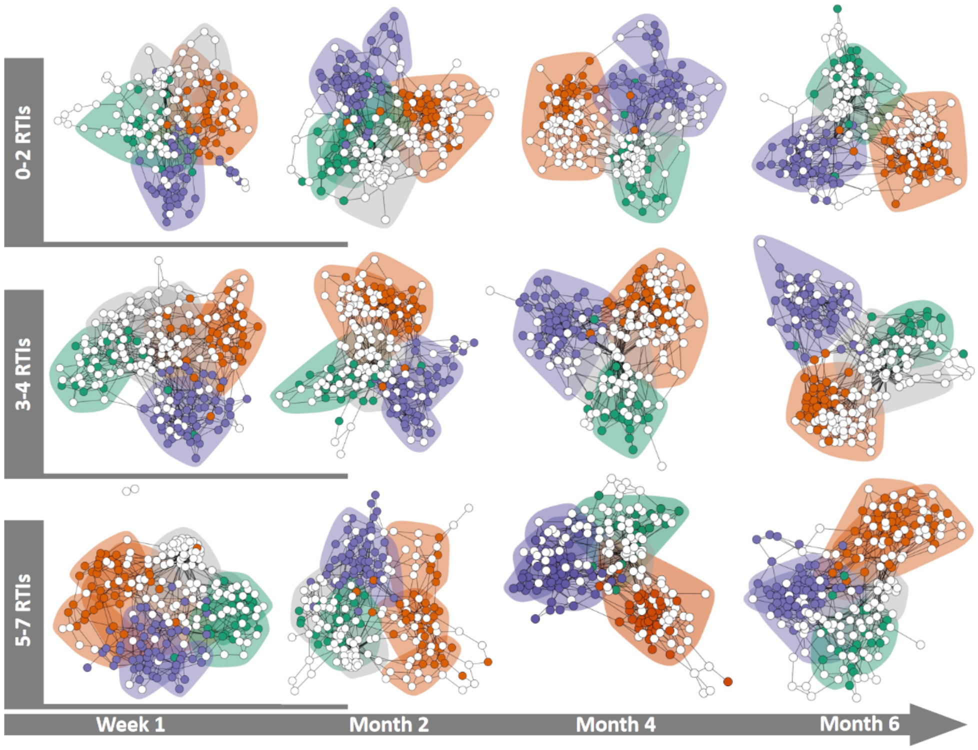

Supplementary Figure 4: Mean cluster similarity split by cluster specificity. The mean cluster similarity is shown for all clusters per RTI group across time split by cluster specificity. The number of faecal clusters was n=18 (n=5 for 0-2 RTIs, n=6 for 3-4 RTIs and n=7 for 5-7 RTIs), n=18 for nasopharyngeal clusters (n=6 for 0-2 RTIs, n=6 for 3-4 RTIs and n=6 for 5-7 RTIs), n=23 for saliva clusters (n=7 for 0-2 RTIs, n=6 for 3-4 RTIs and n=10 for 5-7 RTIs) and n=13 for mix clusters (n=4 for 0-2 RTIs, n=4 for 3-4 RTIs and n=5 for 5-7 RTIs). Boxplots with medians are shown; the lower and upper hinges correspond to the first and third quartiles (the 25th and 75th percentiles); the upper and lower whiskers extend from the hinge to the largest and smallest value no further than 1.5*IQR from the hinge.

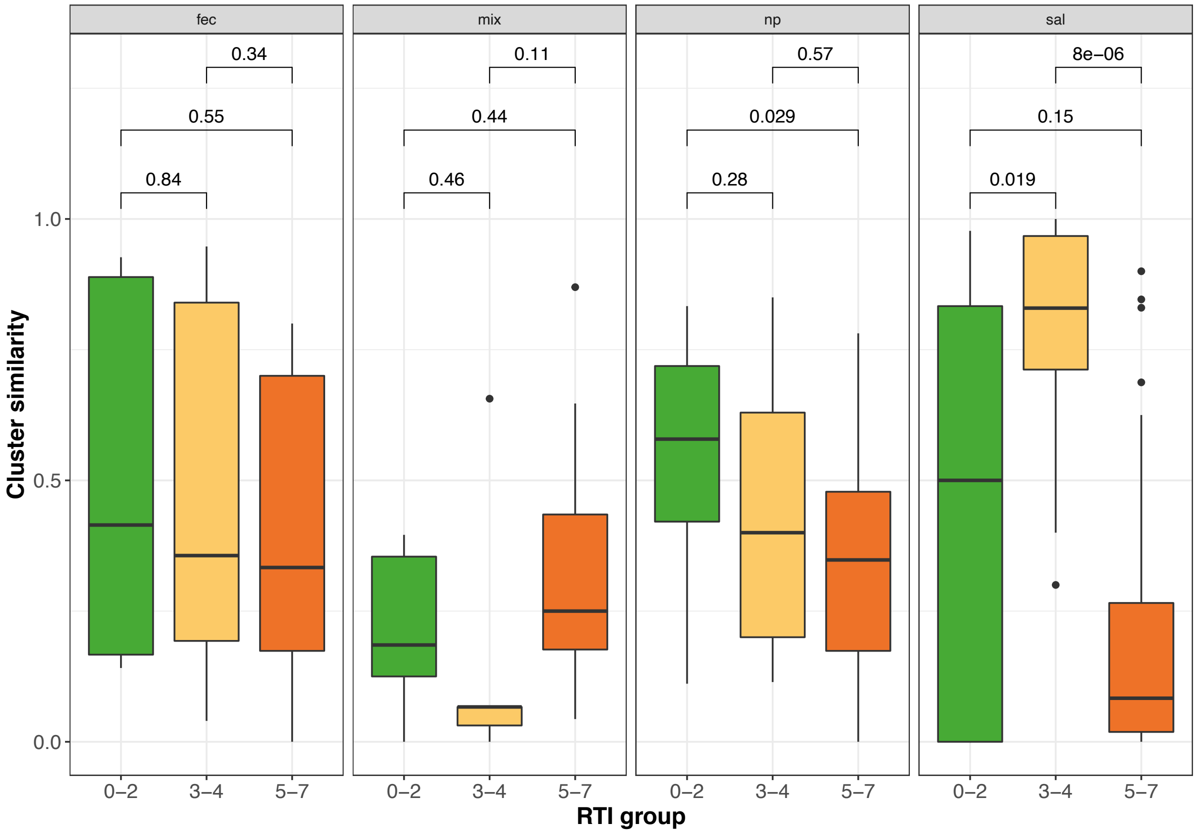

Supplementary Figure 5: Distribution of the number of respiratory tract infections (RTIs) experienced by the study participants over their first year of life. The data is taken from Bosch et. al (2017) and is the same as used in this study. Horizontal dashed lines represent the boundaries for grouping study participants into RTI groups: Green = 0-2 RTIs, yellow = 3-4 RTIs, red = 5-7 RTIs.

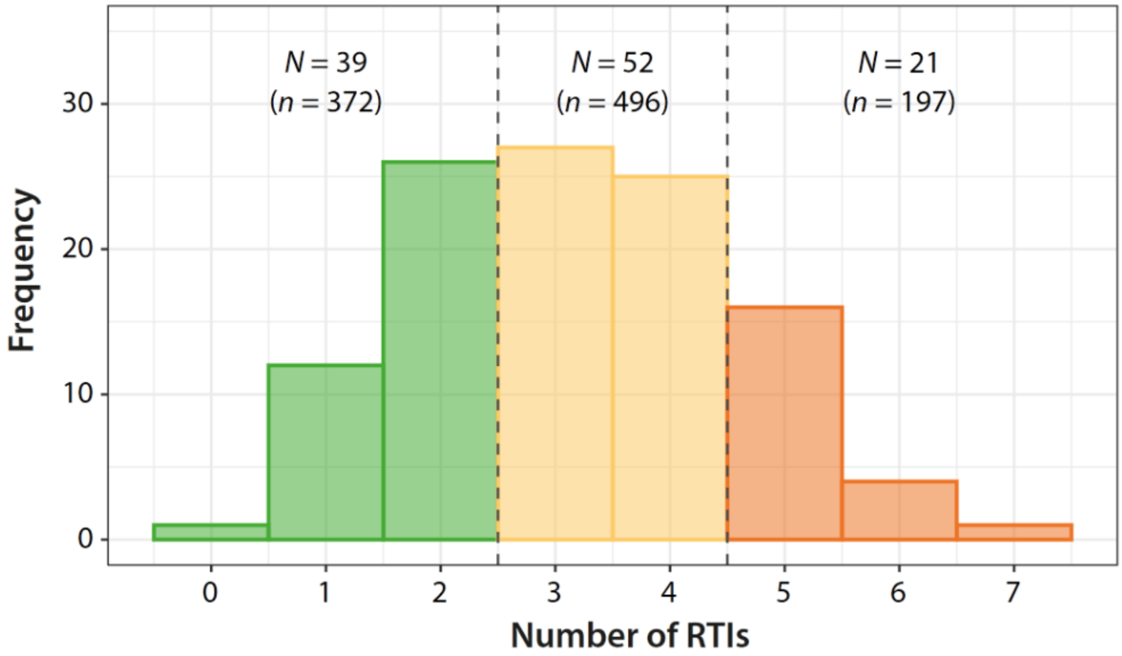

Supplementary Figure 6: Sequencing depth. Rarefaction curves for all samples included in the current study. Lines are coloured by the respiratory tract infection (RTI) group membership of the corresponding sample.

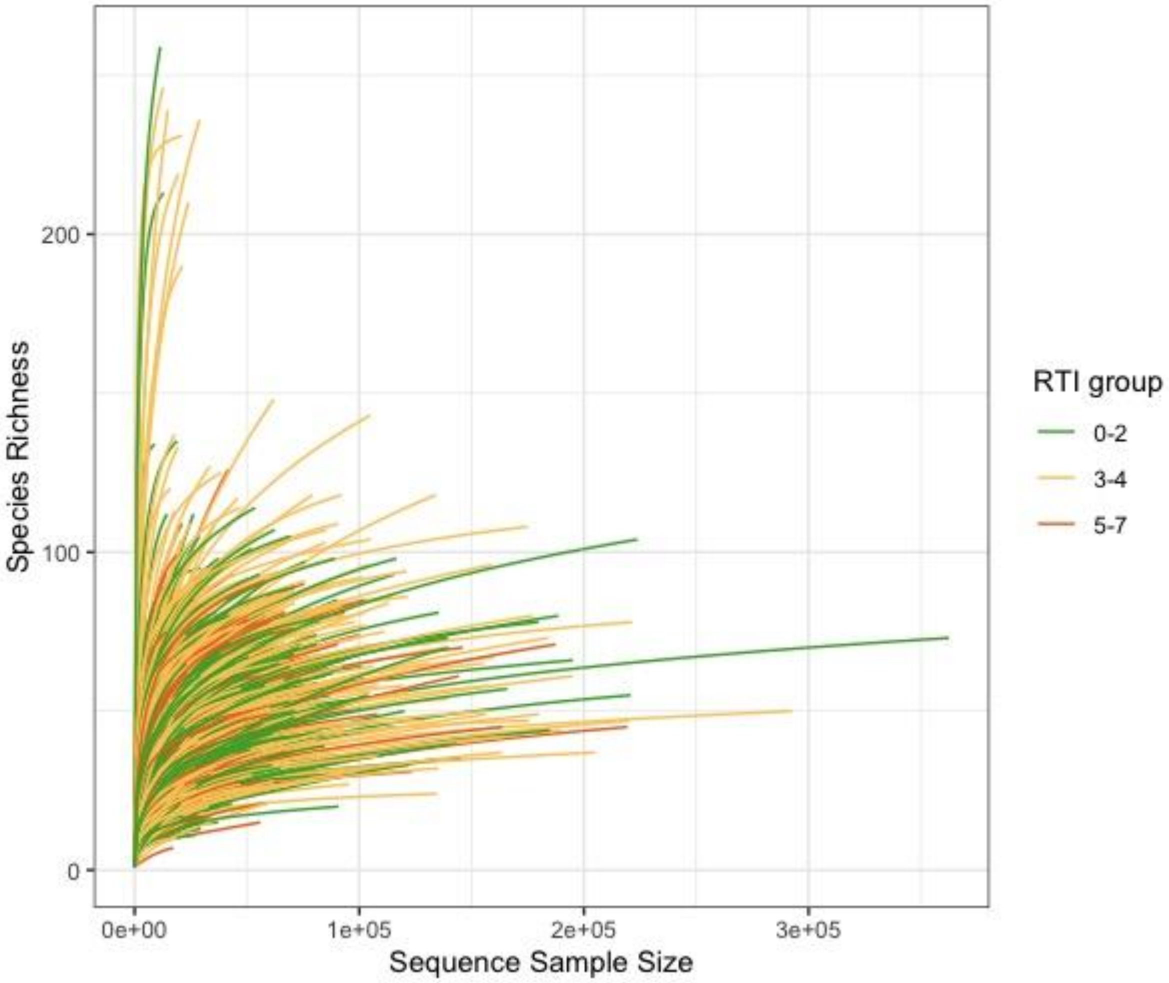

Supplementary Figure 7: Non-metric multidimensional scaling (nMDS) plots, based on Bray-Curtis (BC) dissimilarity between samples, visualising the overall gut microbial community composition cross-sectionally for each niche and time point and stratified by mode of delivery. The ellipses represent the standard deviation of data points belonging to each group, with the centre points of the ellipses calculated using the mean of the coordinates per group.

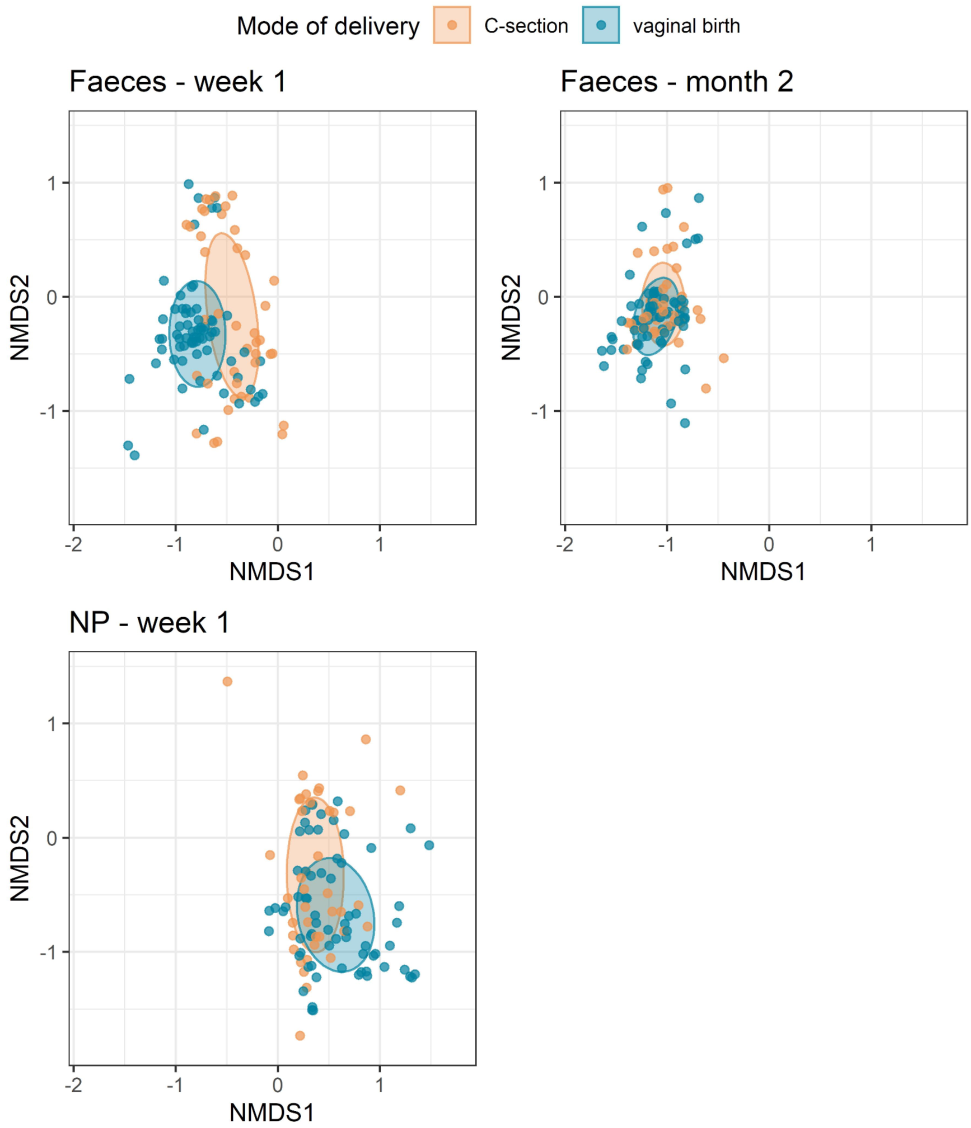

Supplementary Figure 8: Non-metric multidimensional scaling (nMDS) plots, based on Bray-Curtis (BC) dissimilarity between samples, visualising the overall gut microbial community composition cross-sectionally for each niche and time point and stratified by breastfeeding at sampling. The ellipses represent the standard deviation of data points belonging to each group, with the centre points of the ellipses calculated using the mean of the coordinates per group.

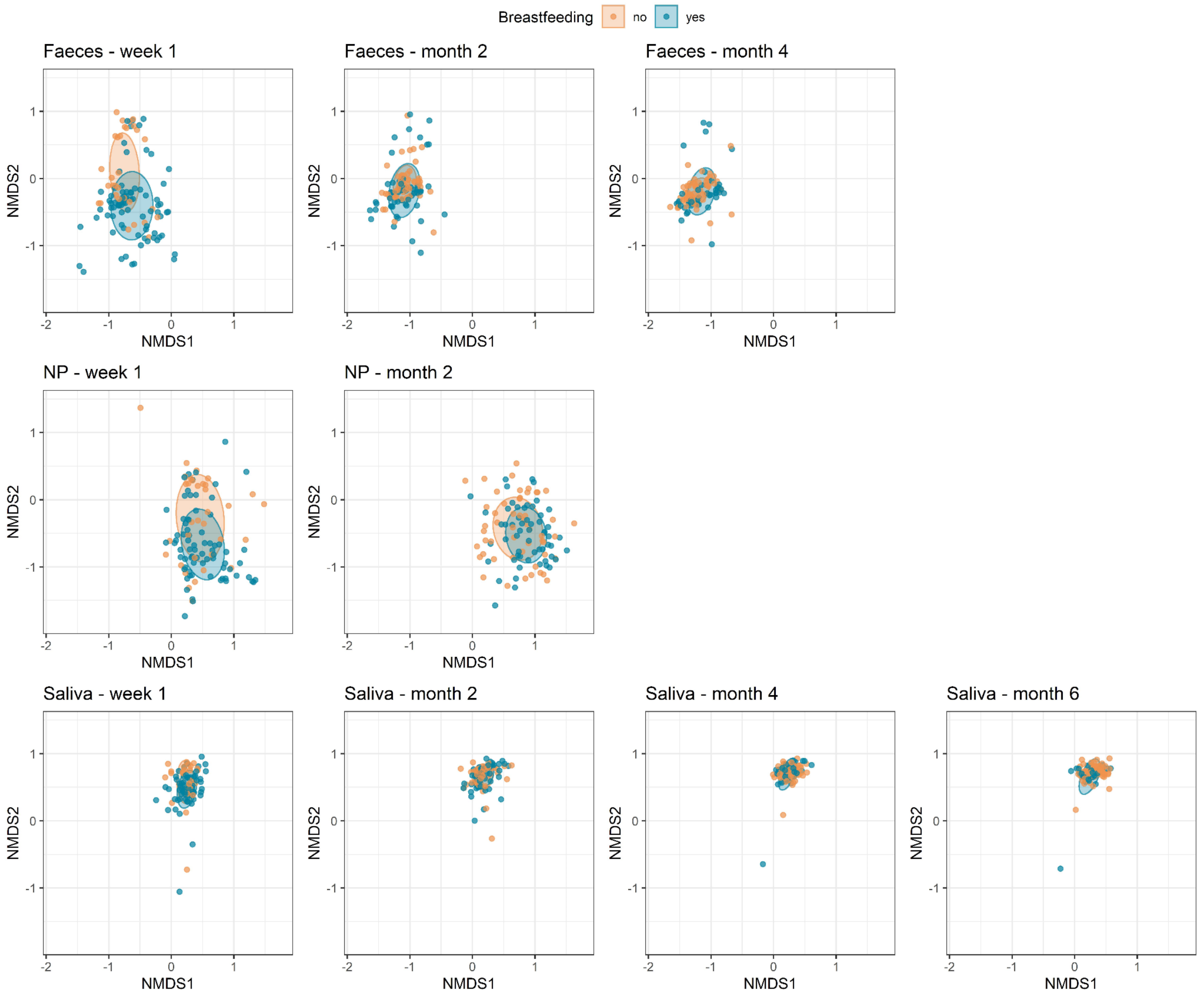

Supplementary Figure 9: Non-metric multidimensional scaling (nMDS) plots, based on Bray-Curtis (BC) dissimilarity between samples, visualising the overall gut microbial community composition cross-sectionally for each niche and time point and stratified by antibiotics treatment 1 month prior to sampling. The ellipses represent the standard deviation of data points belonging to each group, with the centre points of the ellipses calculated using the mean of the coordinates per group.

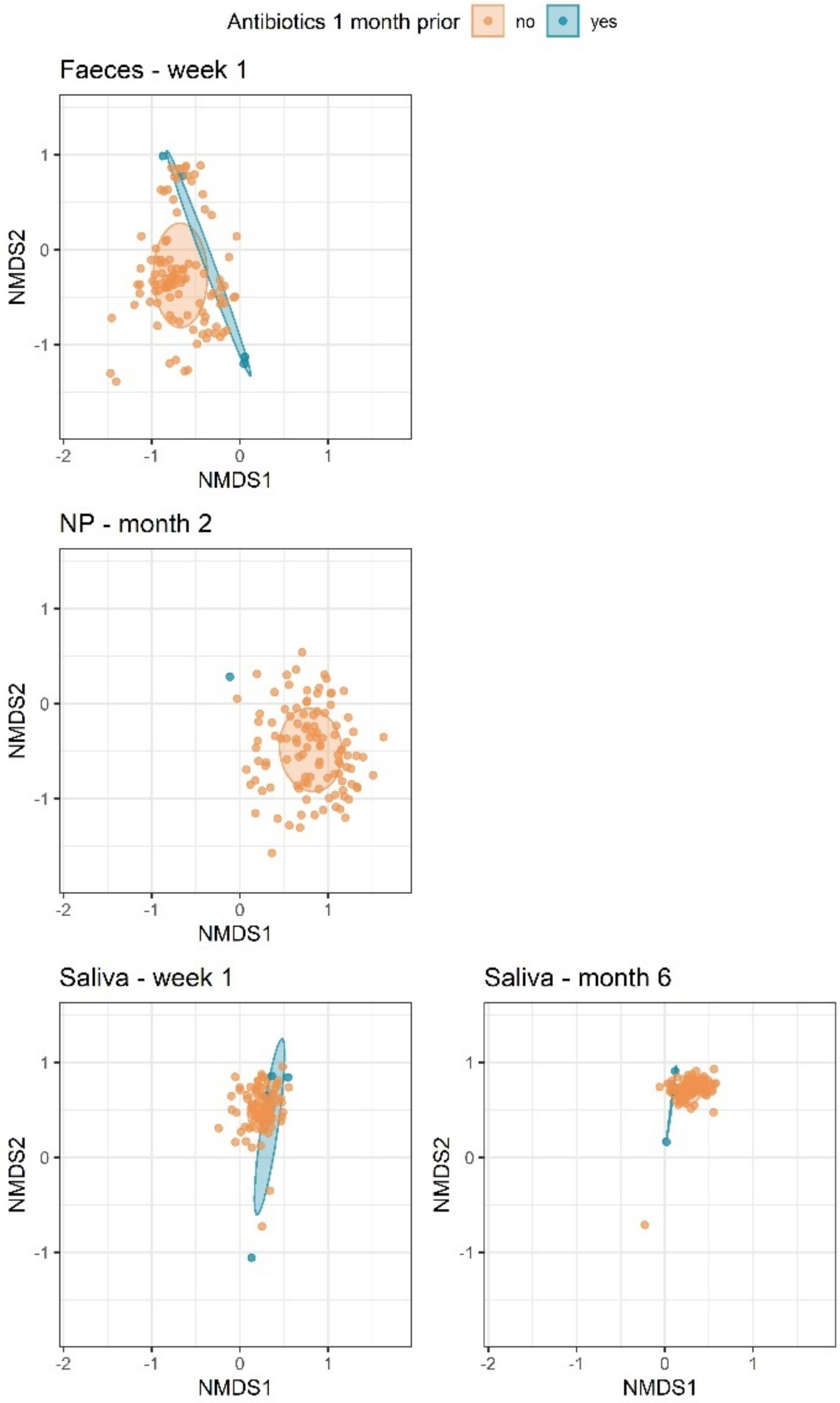

Supplementary Figure 10: Non-metric multidimensional scaling (nMDS) plots, based on Bray-Curtis (BC) dissimilarity between samples, visualising the overall gut microbial community composition cross-sectionally for each niche and time point and stratified by siblings <5 years present in the household. The ellipses represent the standard deviation of data points belonging to each group, with the centre points of the ellipses calculated using the mean of the coordinates per group.

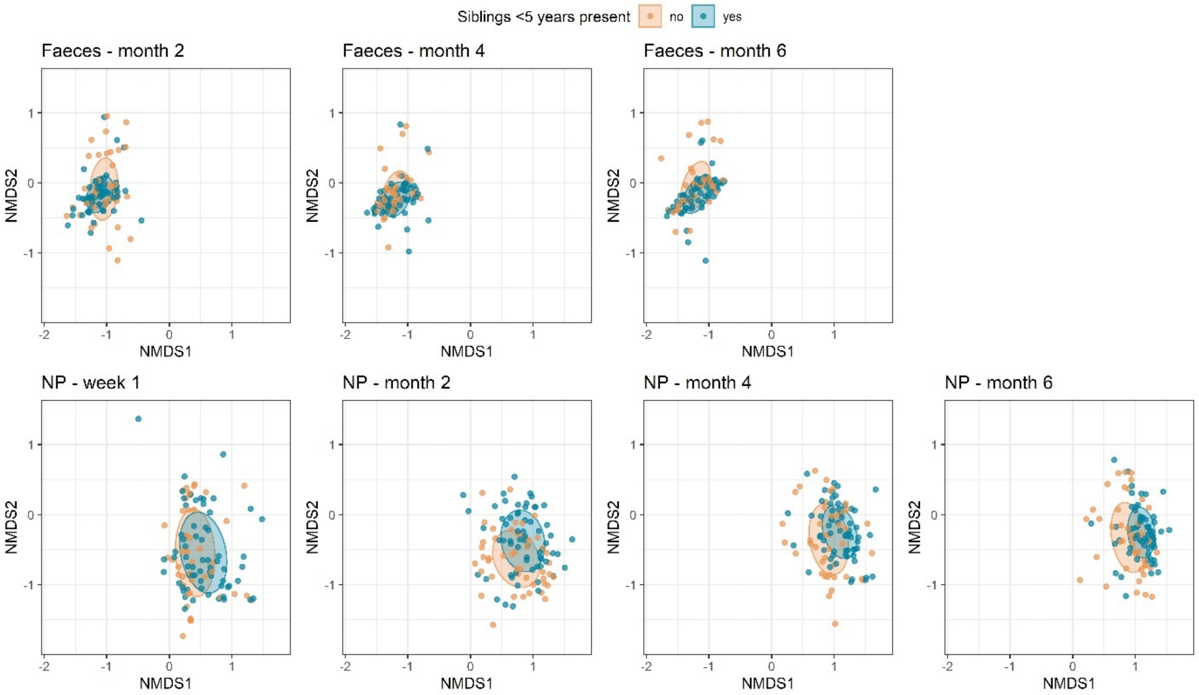

Supplementary Figure 11: Non-metric multidimensional scaling (nMDS) plots, based on Bray-Curtis (BC) dissimilarity between samples, visualising the overall gut microbial community composition cross-sectionally for each niche and time point and stratified by day care attendance. The ellipses represent the standard deviation of data points belonging to each group, with the centre points of the ellipses calculated using the mean of the coordinates per group.

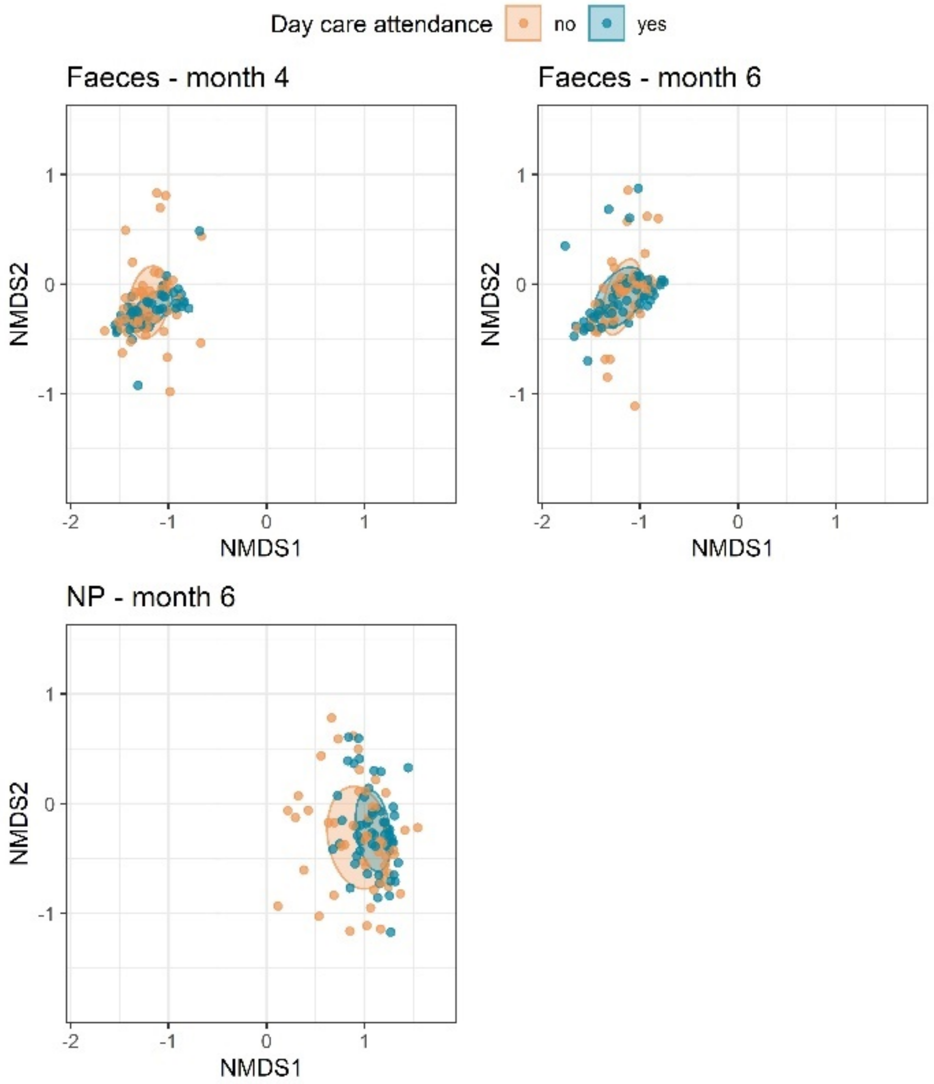

Supplementary Figure 12: Non-metric multidimensional scaling (nMDS) plots, based on Bray-Curtis (BC) dissimilarity between samples, visualising the overall gut microbial community composition cross-sectionally for each niche and time point and stratified by pacifier use. The ellipses represent the standard deviation of data points belonging to each group, with the centre points of the ellipses calculated using the mean of the coordinates per group.

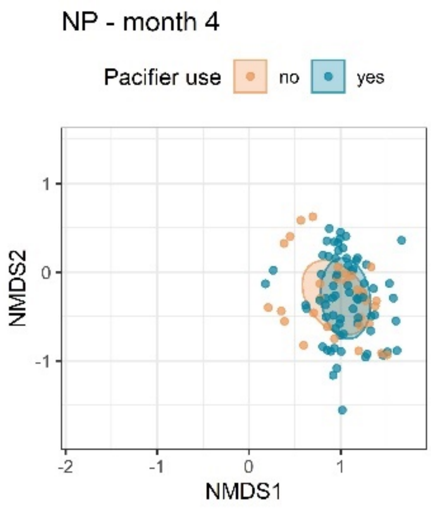

Supplementary Figure 13: Upset plots. Shown are overlapping sets of OTUs between RTI groups (a) and OTUs identified as either hub species, niche indicator species or none (regular species, b).

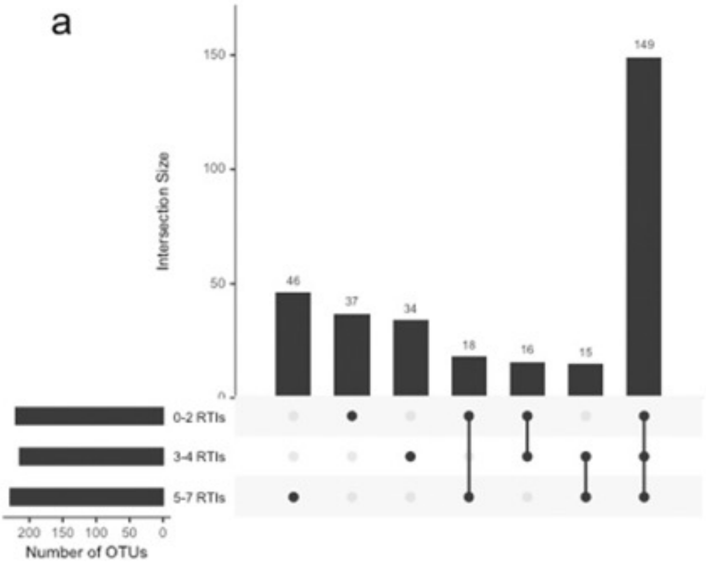

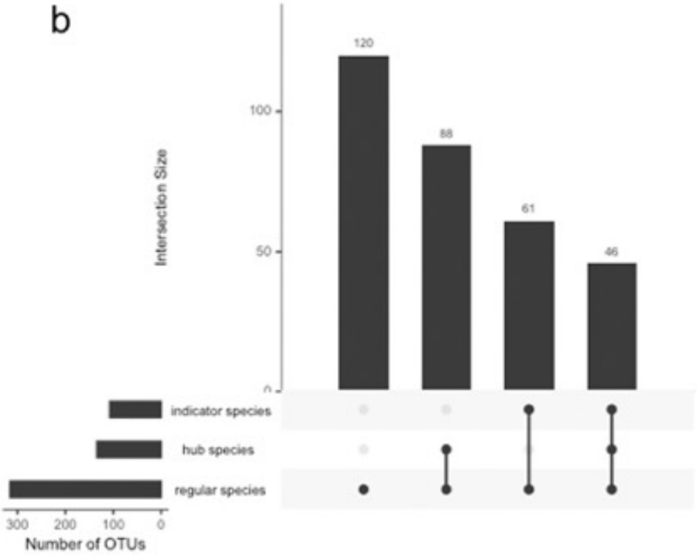

Supplementary Figure 14: Relationship between step length in walktrap.community() and number of clusters within networks. Shown are mean number of clusters overall (a) and split between RTI groups (b) considering all networks. Further, panel (c) shows mean number of main clusters (only clusters containing 10 or more OTUs) overall and split by RTI group (d). Boxplots with medians are shown; the lower and upper hinges correspond to the first and third quartiles (the 25th and 75th percentiles); the upper and lower whiskers extend from the hinge to the largest and smallest value no further than 1.5*IQR from the hinge.

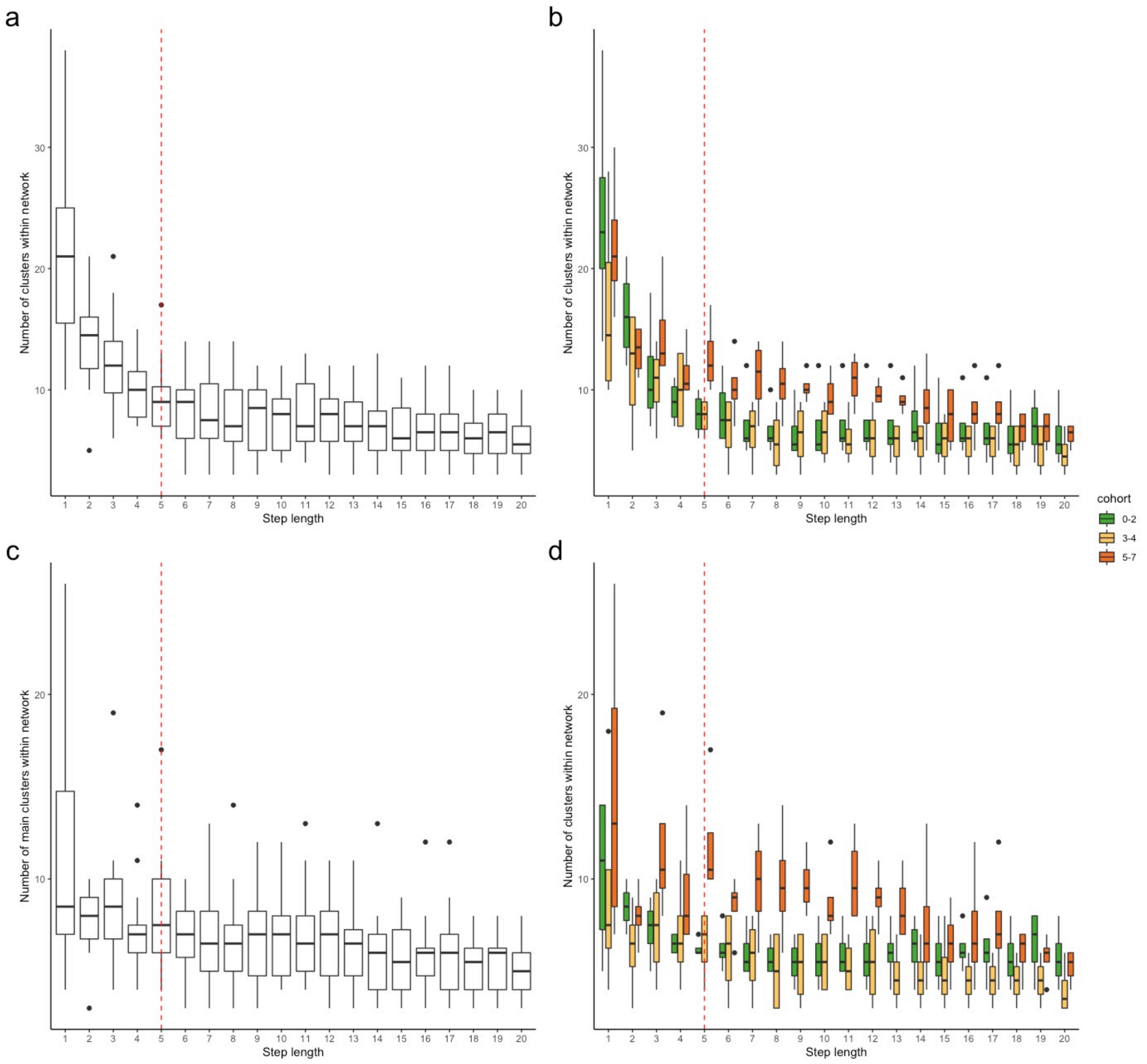

Supplementary Figure 15: Histogram of network cluster sizes split by time point. walktrap.community() was used with step length = 5. The vertical red dashed line represents the threshold of cluster size = 10 above which a cluster was considered a main cluster.

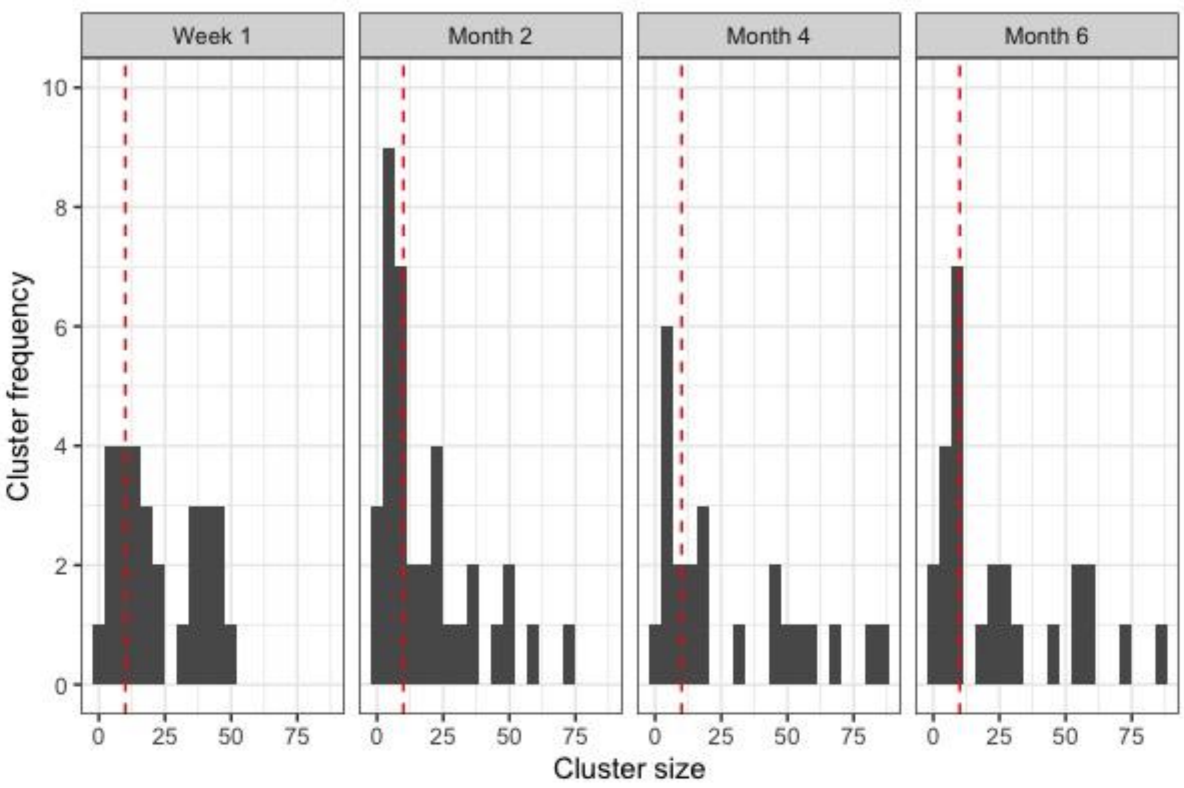

Supplementary Figure 16: Correlation between network metrics. Shown are p-values of Pearson correlation coefficient (top triangle) and a scatterplot (bottom triangle) of each pair of network metrics (betweenness centrality, closeness centrality and degree centrality). The histograms in the diagonal represent the distribution of each network metric within the data. All data are split by RTI group.

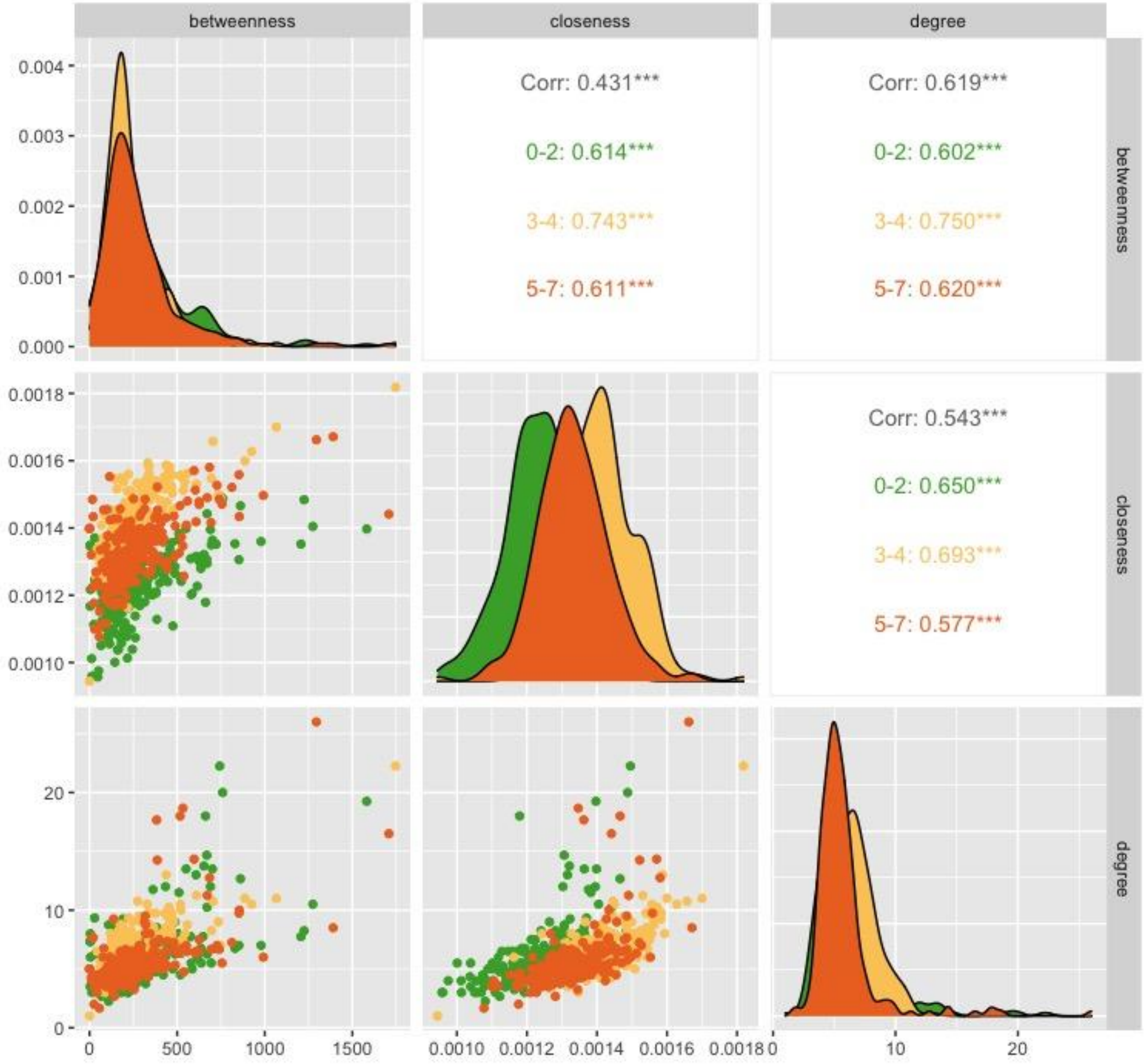

Supplementary Figure 17: Distribution of network metrics for all OTUs in main clusters. Distribution of betweenness centrality values (a), closeness centrality values (b) and degree centrality (c) split by RTI group.

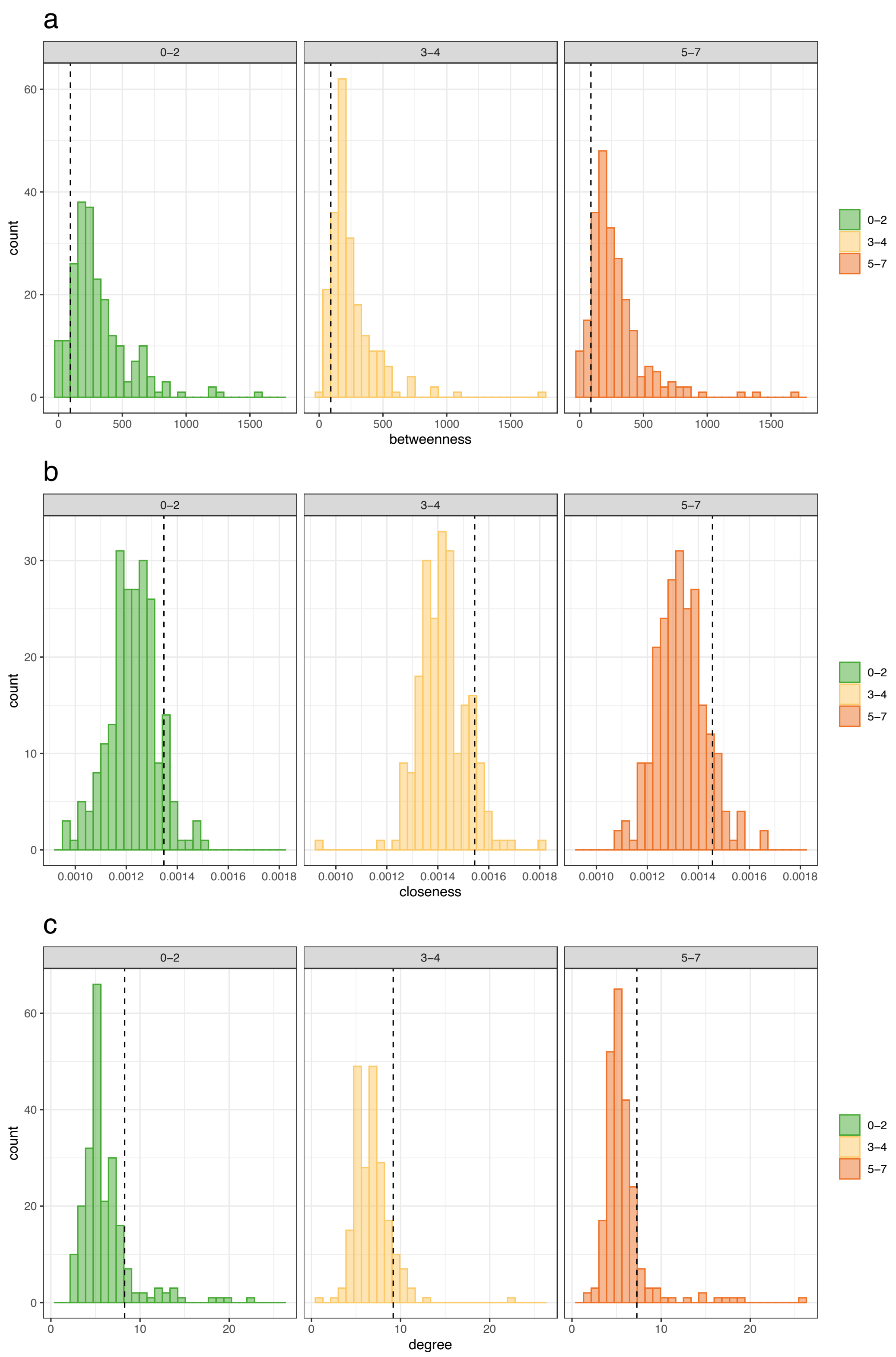

Supplement: Supplementary file 1 — Supplementary information [file 42003_2021_2755_MOESM1_ESM.pdf]
