# Supplementary material for: Microbial community networks across body sites are associated with susceptibility to respiratory infections in infants
Source: Commun Biol. 2021 Oct 28;4:1233. doi: 10.1038/s42003-021-02755-1 (PMC8553847; doi:10.1038/s42003-021-02755-1)
Supplement: Supplementary file 2 — Description of Additional Supplementary Files [file 42003_2021_2755_MOESM2_ESM.pdf]

Description of Additional Supplementary Files

File name: Supplementary Data 1

Description: Tables in Excel format containing the Source Data for the plots in the main manuscript and the Supplementary Information.
