# Supplementary material for: Microbial community networks across body sites are associated with susceptibility to respiratory infections in infants
Source: Commun Biol. 2021 Oct 28;4:1233. doi: 10.1038/s42003-021-02755-1 (PMC8553847; doi:10.1038/s42003-021-02755-1)
Supplement: Supplementary file 4 — Reporting Summary [file 42003_2021_2755_MOESM4_ESM.pdf]

nature portfolio

Corresponding author(s): *Double-blind peer review submissions: write DBPR and your manuscript number here instead of author names.*

Last updated by author(s): *YYYY-MM-DD*

Reporting Summary

Nature Portfolio wishes to improve the reproducibility of the work that we publish. This form provides structure for consistency and transparency in reporting. For further information on Nature Portfolio policies, see our Editorial Policies and the Editorial Policy Checklist.

Statistics

For all statistical analyses, confirm that the following items are present in the figure legend, table legend, main text, or Methods section.

n/a	Confirmed	
☐	☒	The exact sample size (*n*) for each experimental group/condition, given as a discrete number and unit of measurement
☐	☒	A statement on whether measurements were taken from distinct samples or whether the same sample was measured repeatedly
☐	☒	The statistical test(s) used AND whether they are one- or two-sided<br>*Only common tests should be described solely by name; describe more complex techniques in the Methods section.*
☐	☒	A description of all covariates tested
☒	☐	A description of any assumptions or corrections, such as tests of normality and adjustment for multiple comparisons
☐	☒	A full description of the statistical parameters including central tendency (e.g. means) or other basic estimates (e.g. regression coefficient) AND variation (e.g. standard deviation) or associated estimates of uncertainty (e.g. confidence intervals)
☐	☒	For null hypothesis testing, the test statistic (e.g. *F*, *t*, *r*) with confidence intervals, effect sizes, degrees of freedom and *P* value noted<br>*Give P values as exact values whenever suitable.*
☒	☐	For Bayesian analysis, information on the choice of priors and Markov chain Monte Carlo settings
☒	☐	For hierarchical and complex designs, identification of the appropriate level for tests and full reporting of outcomes
☒	☐	Estimates of effect sizes (e.g. Cohen's *d*, Pearson's *r*), indicating how they were calculated

Our web collection on statistics for biologists contains articles on many of the points above.

Software and code

Policy information about availability of computer code

Data collection	Glean Study Manager™ was used to build a database for data collection. This is based on FAB™ version 2.10.1, developed by Sidekick-IT.
Data analysis	Analyses regarding the microbiota composition have been performed in R version 3.4.3, while network analyses have been performed in R versions and 3.6.2

For manuscripts utilizing custom algorithms or software that are central to the research but not yet described in published literature, software must be made available to editors and reviewers. We strongly encourage code deposition in a community repository (e.g. GitHub). See the Nature Portfolio guidelines for submitting code & software for further information.

Data

Policy information about availability of data

All manuscripts must include a data availability statement. This statement should provide the following information, where applicable:

- Accession codes, unique identifiers, or web links for publicly available datasets
- A description of any restrictions on data availability
- For clinical datasets or third party data, please ensure that the statement adheres to our policy

Raw sequence data underlying the findings of this study have been previously deposited in the NCBI Sequence Read Archive (SRA) database as part of other publications with BioProject IDs PRJNA353336, PRJNA450937 and PRJNA481243. The results presented in this manuscript are based on a subset of the sequences deposited under these BioProjects. The source data underlying the tables and figures presented in this manuscript are provided in supplementary data 1.

Field-specific reporting

Please select the one below that is the best fit for your research. If you are not sure, read the appropriate sections before making your selection.

- [x] Life sciences
- [ ] Behavioural & social sciences
- [ ] Ecological, evolutionary & environmental sciences

For a reference copy of the document with all sections, see nature.com/documents/nr-reporting-summary-flat.pdf

Life sciences study design

All studies must disclose on these points even when the disclosure is negative.

Sample size	The study had originally been powered based on the abundance and distribution of previously published microbiota data from infants, ensuring a power of 0.8 to detect at least significant differences in alpha and beta diversity between groups, as well as differences in abundance of the 25 most important operational taxonomical units (OTUs), taking into consideration OTUs with high and low variability and abundance and varying effect sizes. This power calculation was later verified by an online (HMP-based) tool. A total of 120 infants were enrolled. For this manuscript we used microbiota data from samples obtained at four different timepoints (week 1, month 2, month 4 and month 6) and from three different niches, namely the gastrointestinal tract (faecal samples), the upper respiratory tract (nasopharyngeal swabs) and the oral cavity (saliva samples). Metadata was available for the first year of life, but matched samples collected in parallel were only available until month 6.
Data exclusions	We excluded all data from patients that were collected after 6 months of life as samples from all three niches were only available until this time point
Replication	The data presented here are based on patient samples and therefore cannot be reproduced multiple times
Randomization	Not applicable due to the observational study design.
Blinding	Not applicable due to the observational study design.

Reporting for specific materials, systems and methods

We require information from authors about some types of materials, experimental systems and methods used in many studies. Here, indicate whether each material, system or method listed is relevant to your study. If you are not sure if a list item applies to your research, read the appropriate section before selecting a response.

Materials & experimental systems

n/a	Involved in the study	
[x]	[ ]	Antibodies
[x]	[ ]	Eukaryotic cell lines
[x]	[ ]	Palaeontology and archaeology
[x]	[ ]	Animals and other organisms
[ ]	[x]	Human research participants
[x]	[ ]	Clinical data
[x]	[ ]	Dual use research of concern

Methods

n/a	Involved in the study	
[x]	[ ]	ChIP-seq
[x]	[ ]	Flow cytometry
[x]	[ ]	MRI-based neuroimaging

Human research participants

Policy information about studies involving human research participants

Population characteristics	The population included in the study were healthy term-born infants of both sexes recruited at a single Dutch hospital at four different time points (week 1, month 2, month 4 and month 6). A full description of the study cohort can be found in Bosch, A. A. T. M. et al. Development of Upper Respiratory Tract Microbiota in Infancy is Affected by Mode of Delivery. EBioMedicine 9, 336–345 (2016).
Recruitment	Pregnant women were recruited for participation by their gynecologist or midwife between September 1st, 2012 and September 12th, 2014. Inclusion criteria were term birth (gestational age > 37 weeks). Exclusion criteria at baseline were major congenital anomalies, severe maternal or neonatal complications during birth, language barrier, intention to move outside the research area, and parents under the age of 18 years.
Ethics oversight	Written informed consent was obtained from parents of all children and the study was approved by METC Noord-Holland (M012-015, NTR3986).

Note that full information on the approval of the study protocol must also be provided in the manuscript.
